# Supplementary material for: Digital mapping of surface turbulence status and aerodynamic stall on wings of a flying aircraft
Source: Nat Commun. 2023 May 16;14:2792. doi: 10.1038/s41467-023-38486-6 (PMC10188437; doi:10.1038/s41467-023-38486-6)
Supplement: Supplementary file 1 — Supplementary Information [file 41467_2023_38486_MOESM1_ESM.pdf]

Supplementary Information

Digital Mapping of Surface Turbulence Status and Aerodynamic Stall on Wings of a Flying Aircraft

Zijie Xu†[1,2], *Leo N.Y. Cao*†[1,2], *Chengyu Li*†[1,2], *Yingjin Luo*†[1,2], *Erming Su*[1,2], *Weizhe Wang*[3], *Wei Tang*[1,2], *Zhaohui Yao**[3], *Zhong Lin Wang**[1,2,4]

[1]CAS Center for Excellence in Nanoscience, Beijing Key Laboratory of Micro-nano Energy and Sensor, Beijing Institute of Nanoenergy and Nanosystems, Chinese Academy of Sciences, Beijing 101400, China.

[2]School of Nanoscience and Technology, University of Chinese Academy of Sciences, Beijing 100049, China.

[3]School of Engineering Science, University of Chinese Academy of Sciences, Beijing 101408, China.

[4]School of Materials Science and Engineering, Georgia Institute of Technology, Atlanta, GA 30332-0245, USA.

*Corresponding author. Email: Zhong Lin Wang (zhong.wang@mse.gatech.edu) and Zhaohui Yao (yaozh@ucas.edu.cn).

†These authors contributed equally to this work.

Supplementary Note 1

DATSS system's fluid dynamics background

Aircraft, one of the greatest inventions in history, the improvement of its safety performance is a process of continuous exploration and progress. From the early days of aviation, stall has been an inherent hazard. Wright brothers designed their aircraft in a “canard” configuration, facilitating an easy and gentle recovery from stall. Scientists have been trying to achieve stall sensing, an important safety factor in flight activities since the last century. In-flight loss of control caused by stall was the largest fatal accident category for large commercial jet airplane accidents worldwide occurring from 2006 through 2015, which resulted in 15 accidents and 1396 total fatalities of a total of 3296 accidents.

For the force analysis of turbulent viscous fluid, the standard form of Navier-Stokes (N-S) equation is given by:

$$\frac{D\vec{V}}{Dt} = \vec{f_b} - \frac{1}{\rho}\nabla p + \frac{\mu}{\rho}\nabla^2\vec{V}, \tag{1}$$

where $\frac{D\vec{V}}{Dt}$ is the inertial force term of the fluid, $\vec{f_b}$ is volume force term, $-\frac{1}{\rho}\nabla p$ is pressure difference term and $\frac{\mu}{\rho}\nabla^2\vec{V}$ is viscous force term. N-S equation is widely used and discussed in fluid dynamics, especially in the field of aviation. The lift and drag values obtained by the aircraft directly determine whether the aircraft stalls or not. The lift and drag values are affected by the pressure (p), density (ρ), airspeed (v), and angle of attack (α/AoA) of the incoming flow, as well as the area (S), viscosity, compressibility, and configuration of the wing. In the aviation field, the lift-to-drag ratio (K) is commonly used to measure the performance of an aircraft. Generally, a large lift-to-drag ratio indicates a better air characteristic of the aircraft. The K is given by:

$$K = \frac{\frac{1}{2}\rho v^2 C_l S}{\frac{1}{2}\rho v^2 C_d S} = \frac{C_l}{C_d}, \tag{2}$$

where C_l and C_d are lift coefficient and drag coefficient. Normally, as the aircraft's

angle of attack increases, the lift-to-drag ratio first increases to a peak and then decreases. When the critical angle of attack is reached and exceeded, the lift-to-drag ratio decreases rapidly, leading to a serious stall and a risk of crash.

Stall is a condition in which the airflow over the main wing separates at a high AoA, preventing the airplane from gaining lift from the wing. Stall depends only on the stall AoA, affected by the airspeed. Deep-seated reason for the separation of the air flow is that the wing of the aircraft has a certain curvature of the surface of wing. In this way, the fluid micelles are subjected to two forces, one is the shear stress of the surface and the other is the pressure difference force. The direction of these two forces and the incoming flow is opposite. When the flow velocity of the fluid micelles near the surface is reduced to close to zero under the action of these two forces, the shear stress also tends to zero, but the pressure difference force is still there, so the fluid micelles may start to move in the opposite direction of incoming flow as reverse flow. When the backward fluid meets the downstream fluid, it will flow away from the surface, so that the fluid in the entire boundary layer is lifted and separated from the surface. This phenomenon in which the boundary layer fluid leaves the surface due to the reverse pressure difference force of the fluid is boundary layer airflow separation. The boundary layer separation makes the airflow no longer adheres to the airfoil flow, which causes a separation zone above the airfoil after the airflow separation point, and a large amount of reverse curling turbulence in the separation zone. The lift of the airfoil is greatly reduced, the K value decreases rapidly, and the aircraft enters a stall state due to the lack of lift. If the stall state is deep, a stall spin occurs.

To sense the occurrence of stalls, existing commercial stall early warning systems use parameters such as AoA and airspeed. They are mainly divided into two categories: pressure and mechanical types. The pressure type calculates the flight AoA through pressure information, such as a differential pressure sensor, while the mechanical type uses the probe rotation to obtain the flight angle of attack, such as wind vane AoA indicator. While they serve aircraft sensing, there are still many problems that need to be improved: i) accuracy of sensors in complex environments: the air flight environment of aircraft is complex and constantly changing, and the critical AoA and

airspeed for stall are not constant parameters. The occurrence of stall is the result of airflow separation, it is not accurate to calculate the occurrence of stall only by AoA and airspeed when the wing surface is polluted, frozen or damaged. Therefore, more sensors are required to be used together, which makes the structure of the existing solution complicated; ii) system size issues: the differential pressure tube and wind vane handle of the existing size are easily damaged in the complex flight environment, the movable wind vane will generate a certain amount of resistance when the aircraft is flying, which affects local area airflow, and for transonic and supersonic aircraft, the aspect ratio of the wind vane creates a local shock wave. And for emerging multi-purpose UAVs, the large size of existing stall sensors cannot be loaded; iii) invasive integration: the sensor of existing program is power supply required and needs to invade the inside of the wing. It can only be deployed in advance in the design stage of the aircraft, and cannot be retrofitted; iv) algorithms and cost issues: existing solutions use back-end algorithms to analyze various parameters, if the algorithm has defects and risks (such as the Boeing 787-MAX8 incident), it will affect flight safety. At the same time, the high cost of construction makes it impossible to carry it in the aircraft design stage. Therefore, the light weight, high accuracy, adaptability to complex flight environment, small size and low cost stall sensing system will change the existing predicament.

The cause of the aircraft aerodynamic stall is the separation of airflow on the wings during flight. This separation causes the K value (Equation 2) to drop rapidly and leads to flight accidents. This stall caused by the airflow separation, which causes a reciprocating turbulence movement at the rear of the wing surface of the aircraft, similar to the form of vortex streets. Based on the above fluid dynamics background, we fabricated a non-invasive and lightweight active system that can sense and warn the pre-stall and during stall of fixed-wing aircraft by employing conjunct signals as provided by triboelectric and piezoelectric effects.

Supplementary Note 2

DATSS system aerodynamics supplement description

In this work, we use the single-electrode working mode for sensing, and a triangularly arranged rectangular hollow alloy steel sheet is used as the counter electrode. In the flow field, due to the combination of Coanda effect, airflow entrainment and self-vibration of airfoil when the aircraft is not stalled, the airflow over the airfoil causes a constant T-signal to be generated stably. During this process, the Coanda effect is a very important flow phenomenon and is the internal cause of the lift problem, using the Coanda effect, the air flow can be consciously induced to generate an air flow velocity greater than the relative air velocity on the upper surface of the triangularly-arranged rectangular hollow alloy steel sheet, just as the wing obtains lift, the steel sheet obtains an upward lift due to the pressure difference between the upper and lower surfaces. At this time, the rising steel sheet sucks the surrounding air into the lower surface due to its light and thin stiffness, in this moment, the air flow rate sucked by the entrainment effect is faster than the upper surface, lead to the steel sheet obtain a downward pressure and moves downward. Then the entrainment effect is reduced, and the Coanda effect makes the steel sheet rise again, under such cycles, the T-signal exists stably. In addition, the self-vibration of airfoil as it moves in the flow field exacerbates the intensity of the T-signal. As involved in Equation 1, the inertial force of the turbulent flow field is determined by the body force, pressure difference and viscous force of the flow field, and the effect of this inertial force allows the T-signal to be applied. As shown in Supplementary Movie 1-5, we used CFD to simulate the four wing surface flow fields between 0° and 24° (AoA). It can be observed from the video that airflow separation can occur when AOA is 12°. This situation becomes more intense at 16°, 20° and 24°, which is also an important reason for the P-signal to produce grades. It is also the standard for judging the depth of the stall. In Supplementary Movie 6, we tracked the aerodynamic stall and recover process of the aircraft by DATSS system in the wind tunnel.

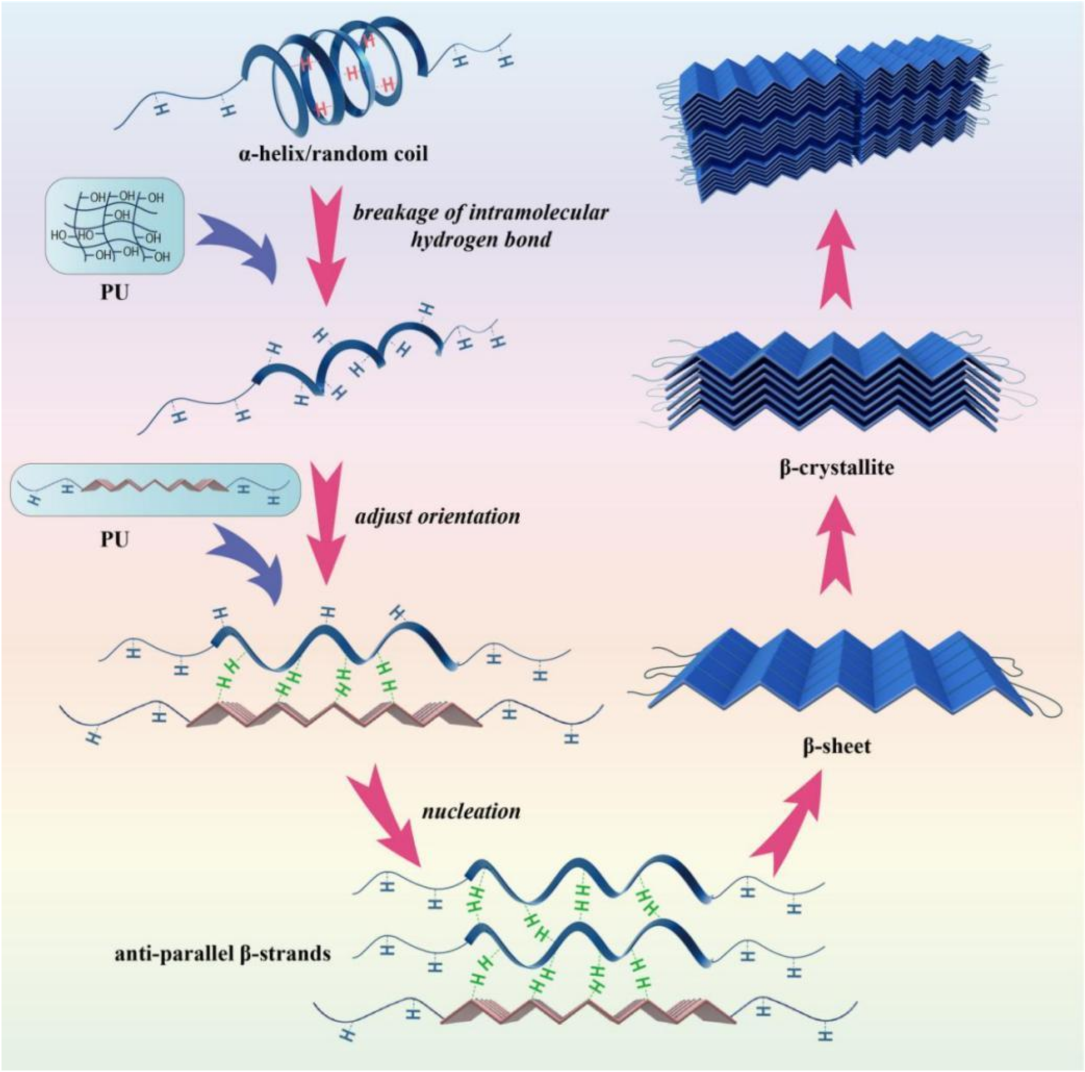

Supplementary Fig. 1 Mechanism of secondary structure transformation of silk fibroin.

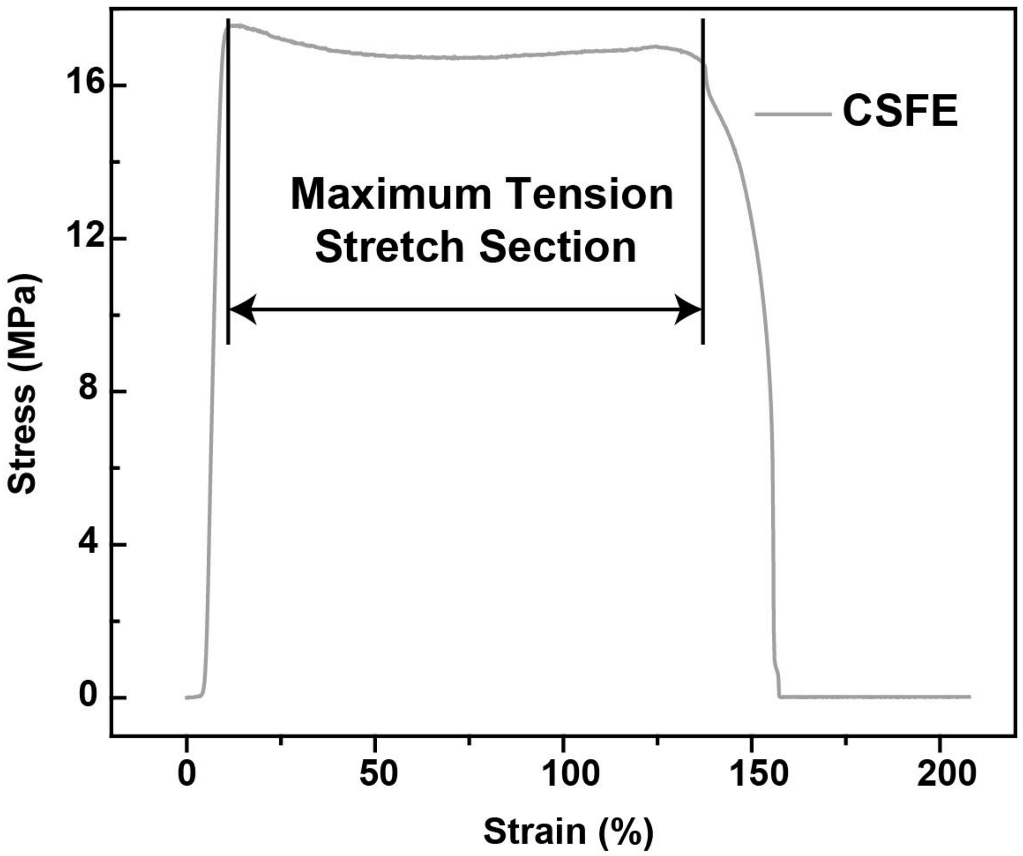

Supplementary Fig. 2 Stress-strain test curve of CSFE.

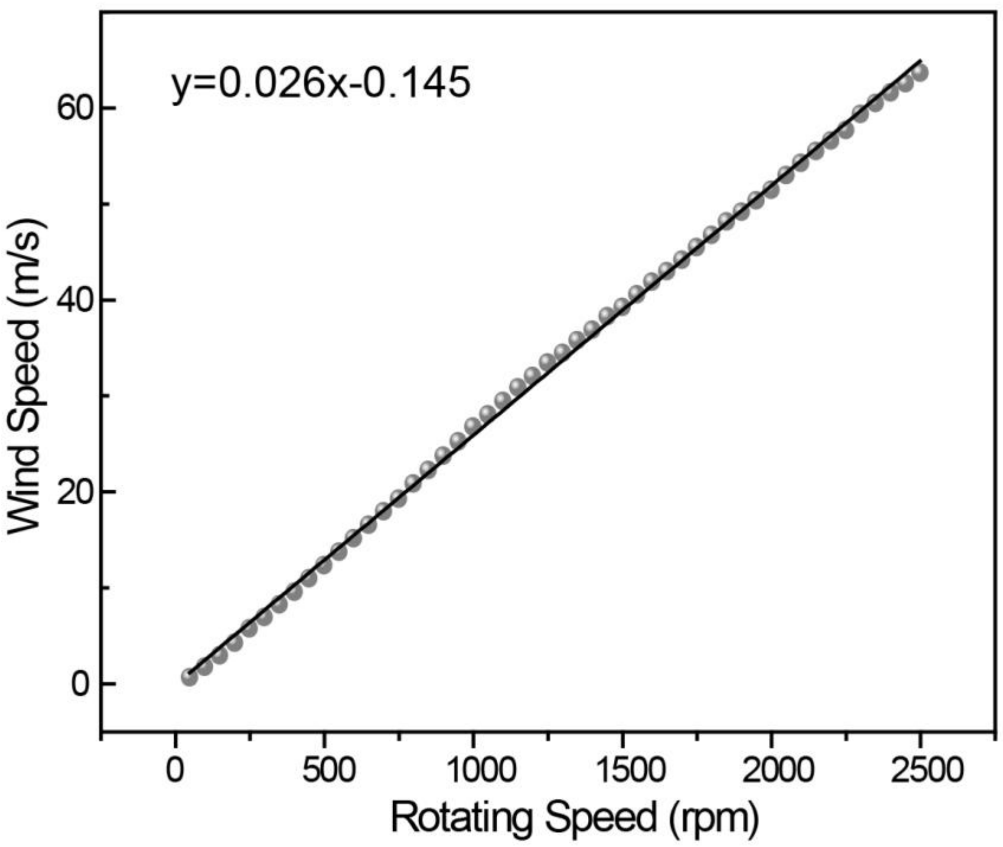

Supplementary Fig. 3 The relationship between motor speed and wind speed in the recirculation wind tunnel.

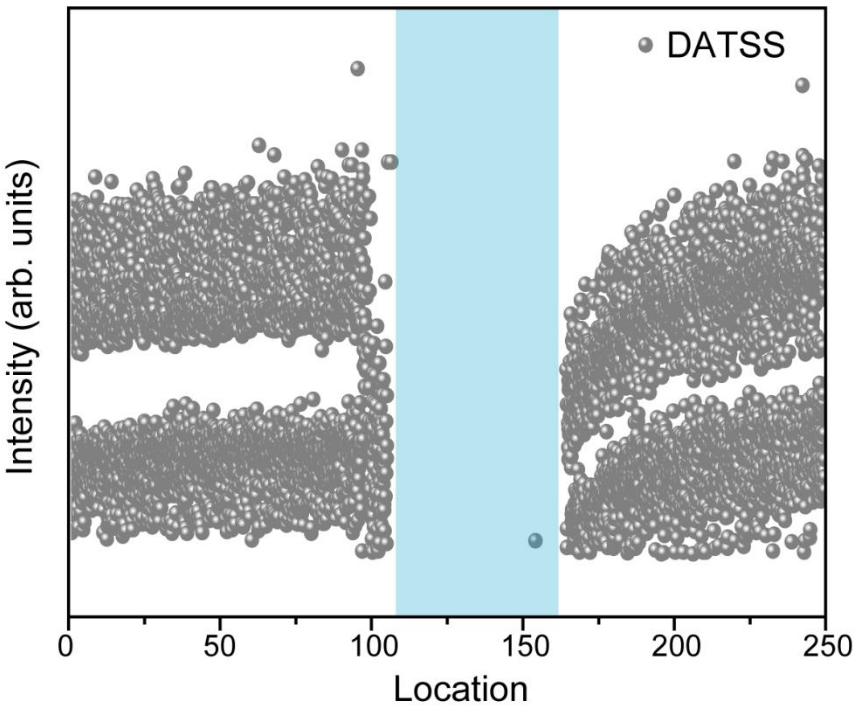

Supplementary Fig. 4 The working condition of T-signal of DATSS system under 60 m/s wind speed.

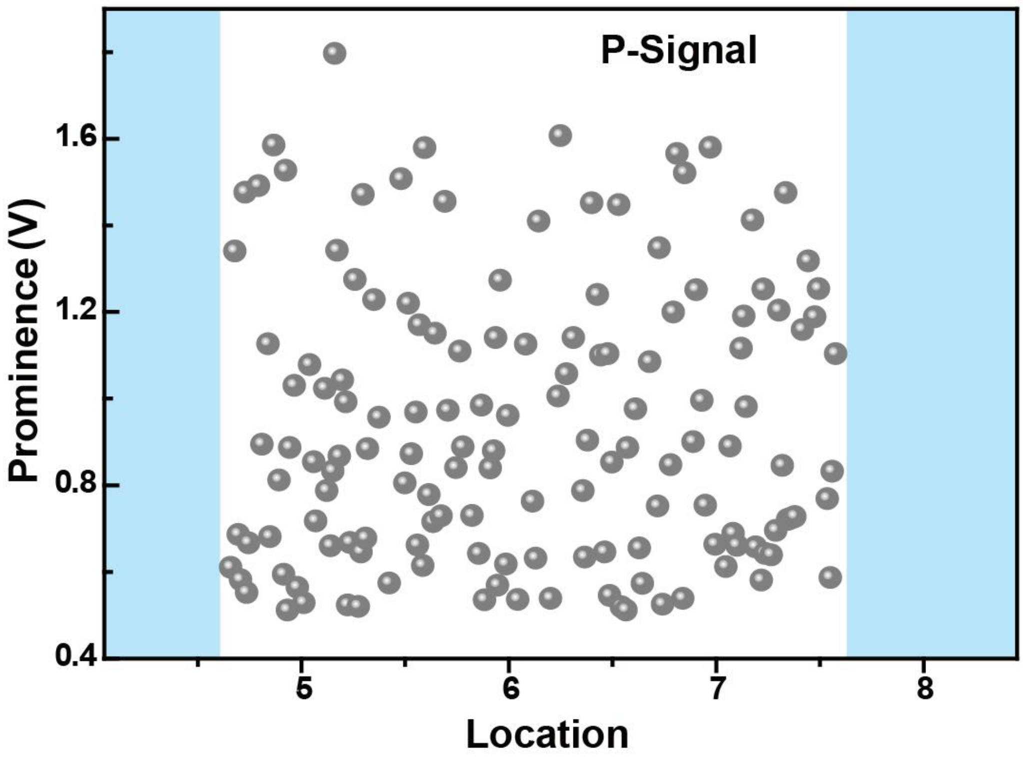

Supplementary Fig. 5 DATSS system stall sensing P-signal after prominence (function of findpeaks in MatLab) processing.

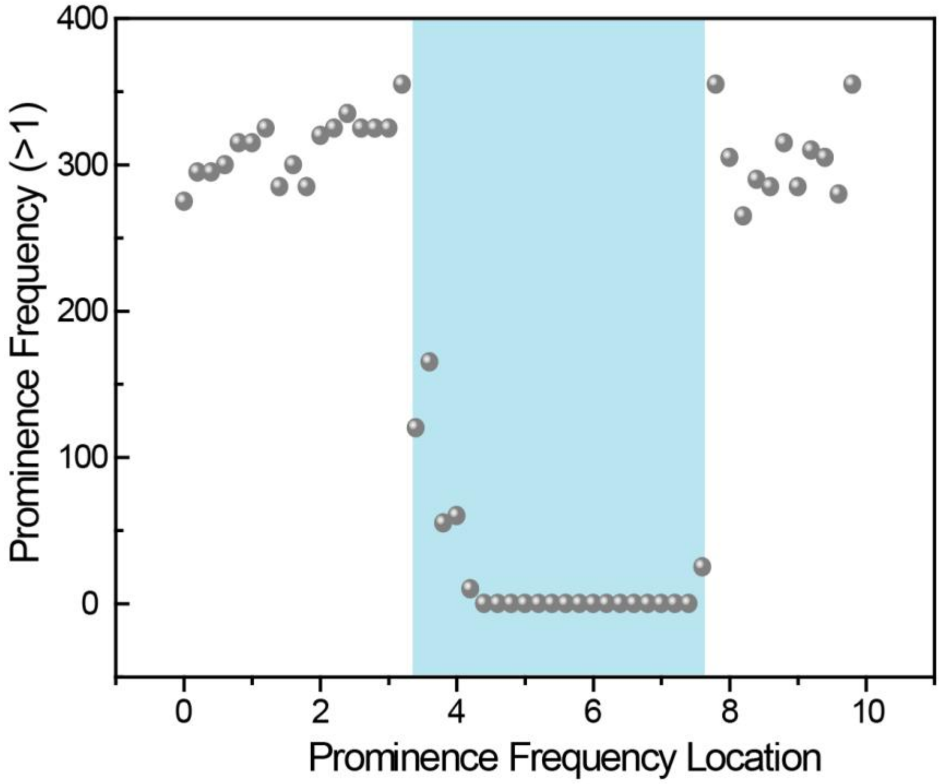

Supplementary Fig. 6 DATSS system stall sensing T-signal after prominence frequency (function of findpeaks in MatLab) processing.

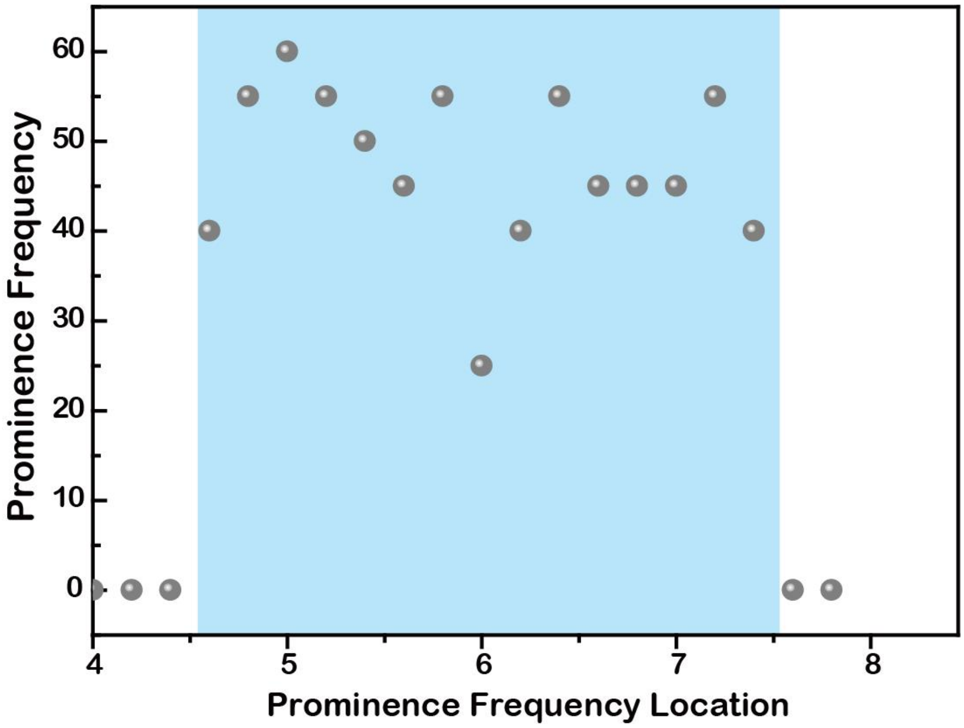

Supplementary Fig. 7 DATSS system stall sensing P-signal after prominence frequency (function of findpeaks in MatLab) processing.

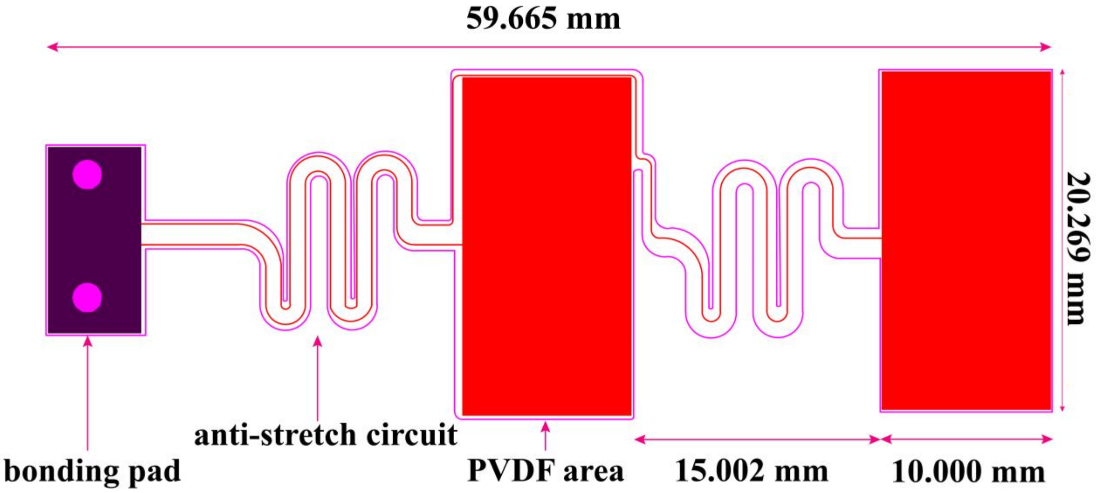

Supplementary Fig. 8 Flexible PCB design for graded P-signals.

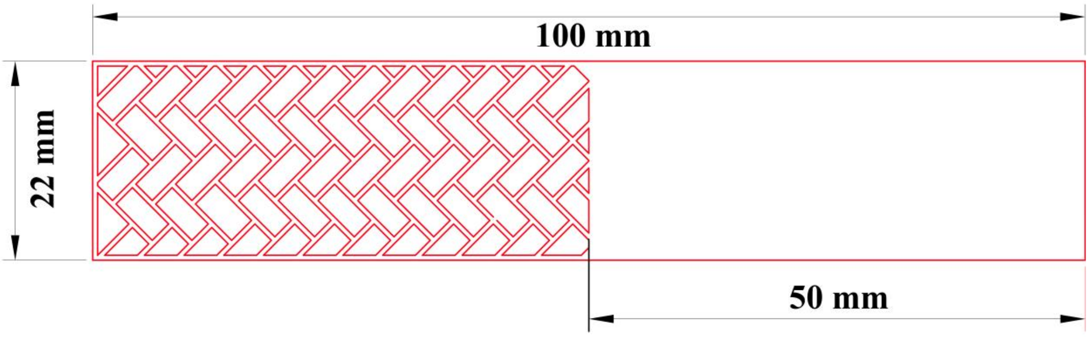

Supplementary Fig. 9 Structural design drawing of hollow alloy steel sheet.

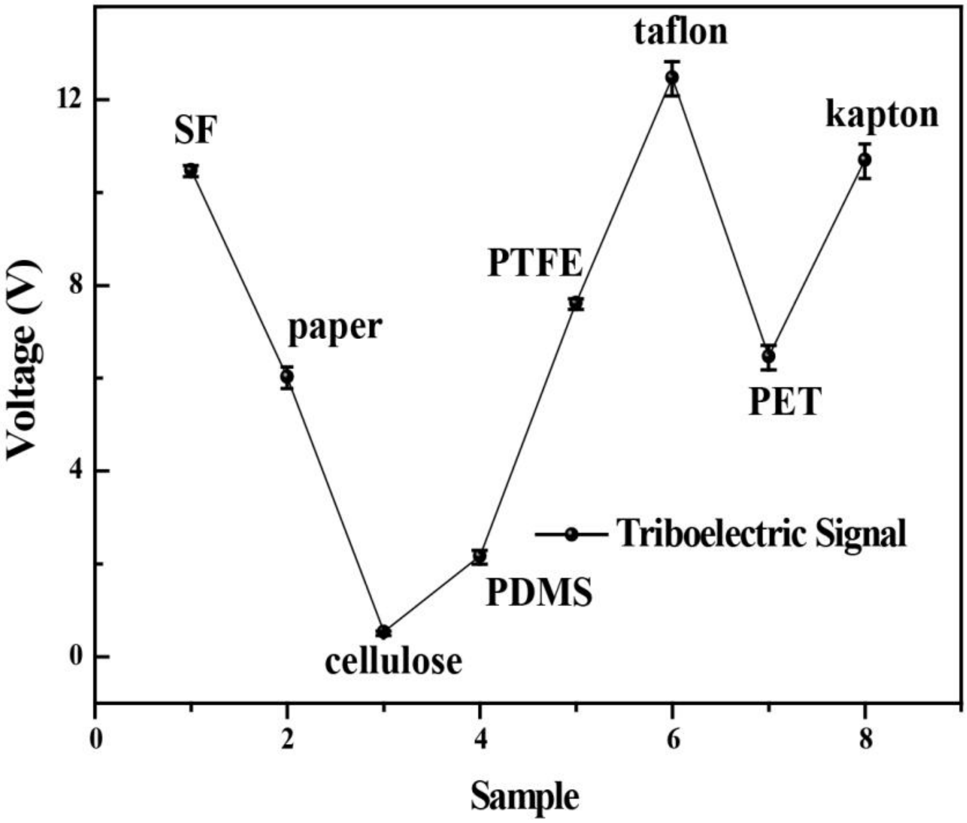

Supplementary Fig. 10 Comparison of T-signal of hollow alloy steel sheet material coupled with different materials.

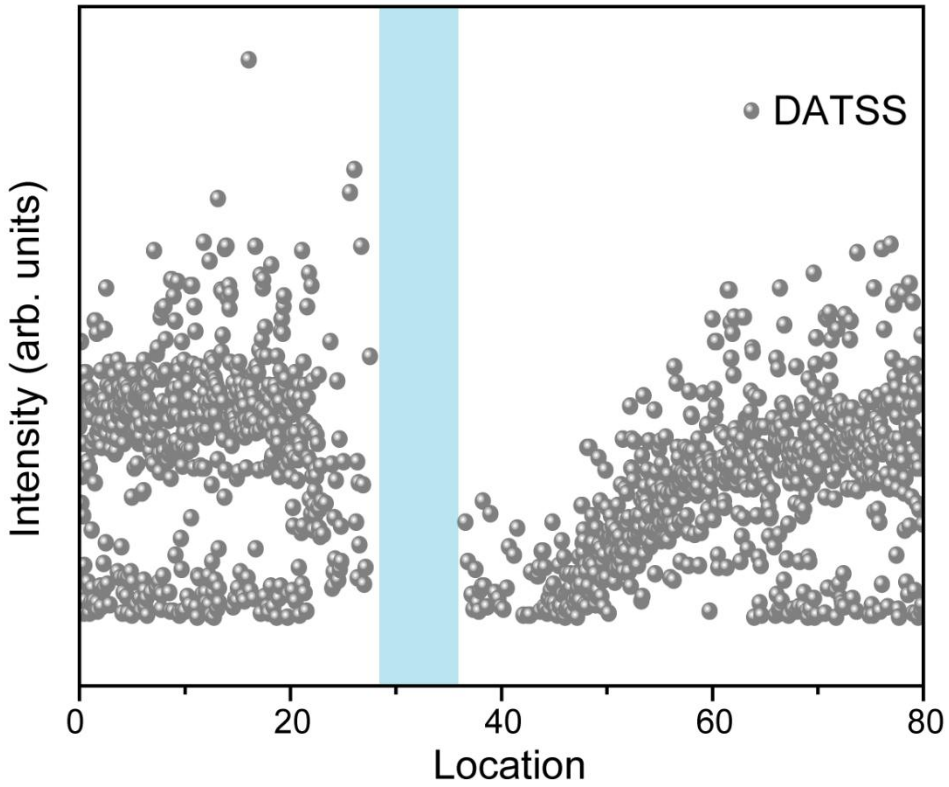

Supplementary Fig. 11 Stall sensing T-signal of DATSS system under surface icing condition.

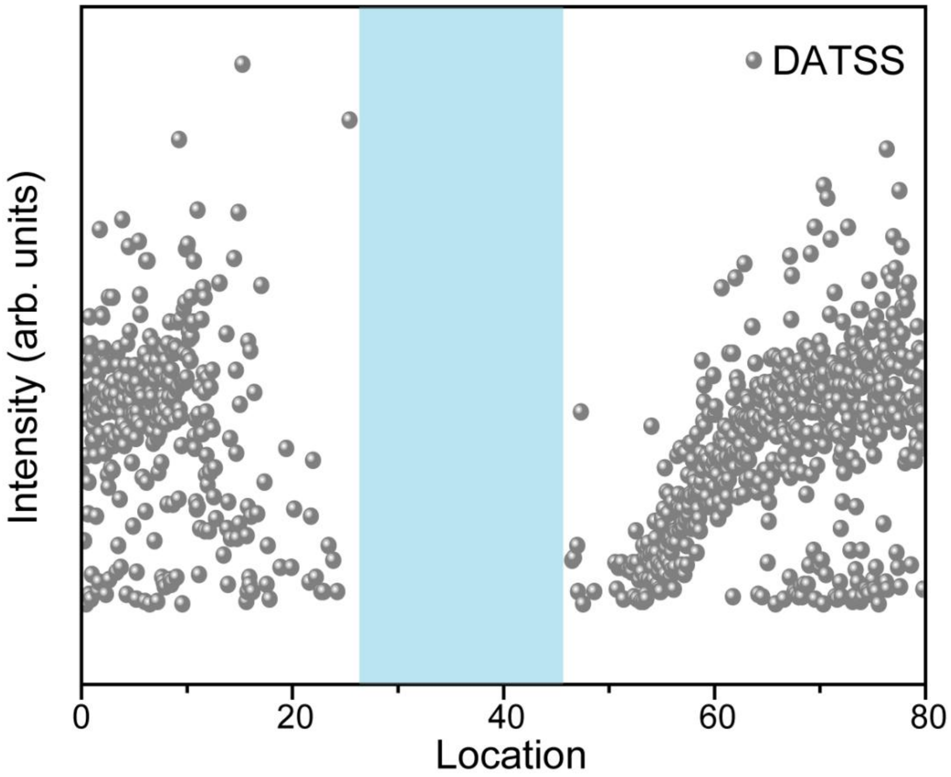

Supplementary Fig. 12 Stall sensing T-signal of DATSS system in rain condition.

Supplementary Table 1 Wind tunnel test used airfoil parameters.

wind tunnel airfoil parameters					
x/c	y/c	c	x	yup	ydown
0.0000	0.0000	200.0000	0.0000	0.0000	0.0000
0.0125	0.0189	200.0000	2.5000	3.7800	-3.7800
0.0250	0.0262	200.0000	5.0000	5.2400	-5.2400
0.0500	0.0356	200.0000	10.0000	7.1200	-7.1200
0.0750	0.0420	200.0000	15.0000	8.4000	-8.4000
0.1000	0.0468	200.0000	20.0000	9.3600	-9.3600
0.1500	0.0535	200.0000	30.0000	10.7000	-10.7000
0.2000	0.0574	200.0000	40.0000	11.4800	-11.4800
0.3000	0.0600	200.0000	60.0000	12.0000	-12.0000
0.4000	0.0580	200.0000	80.0000	11.6000	-11.6000
0.5000	0.0529	200.0000	100.0000	10.5800	-10.5800
0.6000	0.0456	200.0000	120.0000	9.1200	-9.1200
0.7000	0.0366	200.0000	140.0000	7.3200	-7.3200
0.8000	0.0262	200.0000	160.0000	5.2400	-5.2400
0.9000	0.0145	200.0000	180.0000	2.9000	-2.9000
0.9500	0.0081	200.0000	190.0000	1.6200	-1.6200
1.0000	0.0000	200.0000	200.0000	0.0000	0.0000

Supplementary Table 2 Calculation of Reynolds number at different speeds in wind tunnel test.

Re	558659.2	837988.8
V (m/s)	40	60
ρ (kg/m^3)	1.250	1.250
η (Pa·s)	0.179×10^{-4}	0.179×10^{-4}
d (mm)	200	200

Supplementary Note 3

Mechanics principle of T/P-signal generation of DATSS system

In this part, we analyzed the force in the working process of the DATSS system to better help us understand and optimize the design of the DATSS system (Supplementary Fig. 13). During the entire working process of the DATSS system, due to the Coanda effect, airflow entrainment, self-vibration of airfoil and stall turbulent airflow separation, there were four kinds of forces that affected the DATSS system: gravity, elasticity, wind thrust and differential pressure. First, we analyzed the force of the T-signal during the generation process. When the airfoil separation of the aircraft did not occur, due to the influence of the Coanda effect, the steel sheet in the DATSS system was first affected by the upward pressure differential force and elastic force, and started to move upward. At the same time, the steel sheet was also affected by gravity and wind thrust effect. When the steel sheet moved up to a certain height, the direction of the elastic force was reversed. At this time, due to the reverse elastic force and the entrainment effect, the elastic force and the differential pressure force were in the opposite direction, together with the action of gravity and wind thrust, the steel sheet moved downward. When the steel sheet moved up and down repeatedly, the T-signal was continuously generated. For the force condition of the P-signal, the difference from the T-signal was that the wind thrust changed to the opposite direction due to the separation of airflow on the airfoil surface after stalling. Therefore, under the action of the reverse wind thrust, the P-signal was continuously generated.

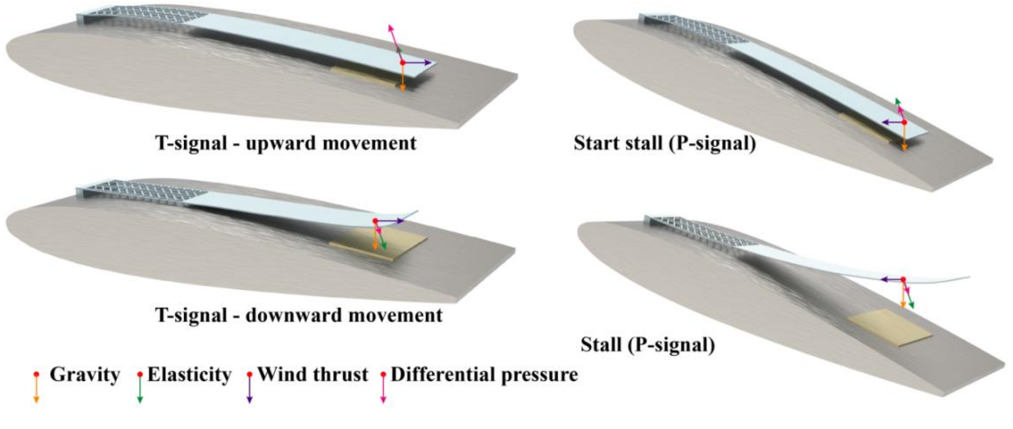

Supplementary Fig. 13 Analysis of mechanics principle of T/P-signal generation of DATSS system.

Supplementary Note 4

Discussion on the influence of DATSS system on lift and drag of aircraft airfoil

In this part, we discuss the influence of DATSS system on lift and drag of aircraft airfoil. As the sensor is based on fluttering motion, it would naturally affect the efficiency of the aircraft by introducing new friction and drag to the wings. When designing the DATSS system, we have fully considered the impact on the flight efficiency of the aircraft. For the influence of the flight efficiency of the aircraft, two factors are mainly considered, lift and drag.

(1) For drag, the introduction of DATSS system mainly affects friction resistance and interference resistance. The DATSS system should minimize the effects of drag. Firstly, the interference resistance is discussed. DATSS adopts a streamlined design in configuration. The ultra-thin hollow alloy steel sheet can conform to the airfoil of the aircraft and minimize the generation of interference resistance. When the T-signal (triboelectric signal) is working, we optimized the length of the steel sheet to make the vibration amplitude within 1 cm (as shown in Supplementary Video 6, different airfoils can be optimized differently), the height (1 cm) is less than the height of some screws on the airfoil of commercial aircraft. The counter electrode is also thin to produce less interference resistance. Next, we discuss the friction resistance. When designing the DATSS system, in order to reduce the frictional resistance, we selected a stainless steel material with a smooth surface (low friction coefficient), and designed a hollow structure to reduce the surface area and mass of the DATSS system. The quality of the entire hollow alloy steel sheet is 0.9 g. In addition, the stiffness of the steel sheet is low, and its natural state is a streamlined structure that fits the airfoil. This makes it aerodynamically better for airflow over the surface.

(2) For lift, DATSS system acts like active vortex generator, that can slightly delay airflow boundary separation and reduce pressure drag. When designing the DATSS system, we referenced a large number of aircraft design literature, from which we conceived a reasonable system structure that can increase a part of the lift (which may have very little impact, we explain from the theoretical side). i) Blocking effect of

reversed air, when the airfoil flow separation starts, the rolled steel sheet can use its rolled-up tail to block the reversely separated airflow while generating the P-signal (piezoelectric signal), so that this part of the airflow can be redirected back to the incoming flow direction of the aircraft. This blocking effect can play a part in delaying the stall by reducing pressure drag. ii) Recovery airflow effect. In the early stage of airflow separation, the steel sheet that flutters up and down will exert downward force on the airflow below during the descending process, so that this part of the airflow delays the airflow separation from the airfoil (similar to the action of a vortex generator)[1,2] .

(3) Experimental supplement. To verify the above theory, we supplement the relevant experiments of the lift test. The lift force of the model was tested using a six-axis force balance in a recirculation wind tunnel. Six-axis force balance is a standard equipment used to test lift force of aircraft. The device is shown in Supplementary Fig. 14, considering that gravity might cause some interference with the lift test, we installed the model wing perpendicular to the direction in which it operates, so that gravity and lift are perpendicular to each other without interference. We compared and tested the airfoil lift with four DATSS units and without DATSS, as shown in Supplementary Fig. 15. It can be seen from the figure that even if the DATSS system is installed on the whole airfoil in array, it has almost no impact on the airfoil lift, and even more than the original lift at some data points (in the figure, the wind speed in the wind tunnel increased gradually over time, finally, the final lift value was compared).

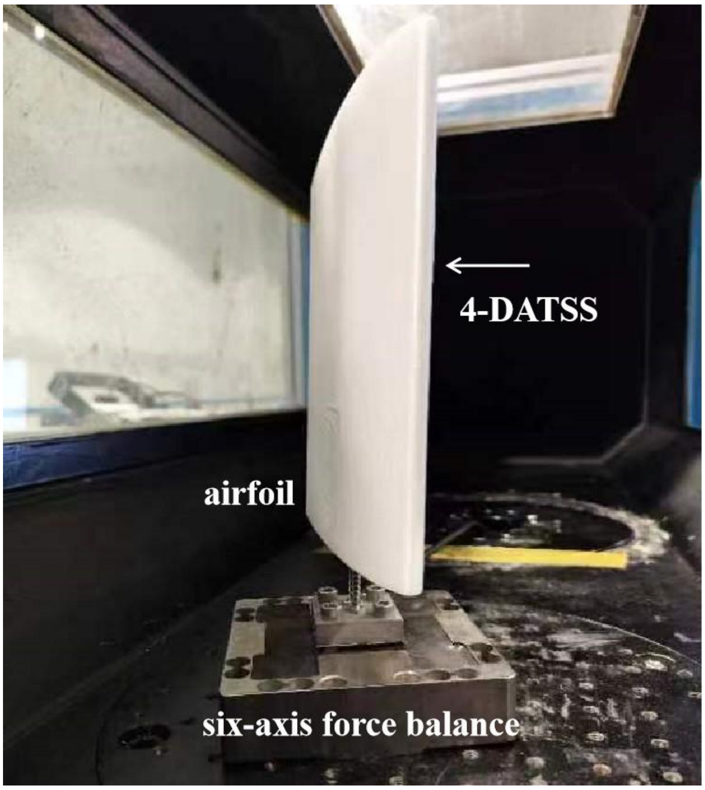

Supplementary Fig. 14 Photograph of the wind tunnel test of the six-axis force balance.

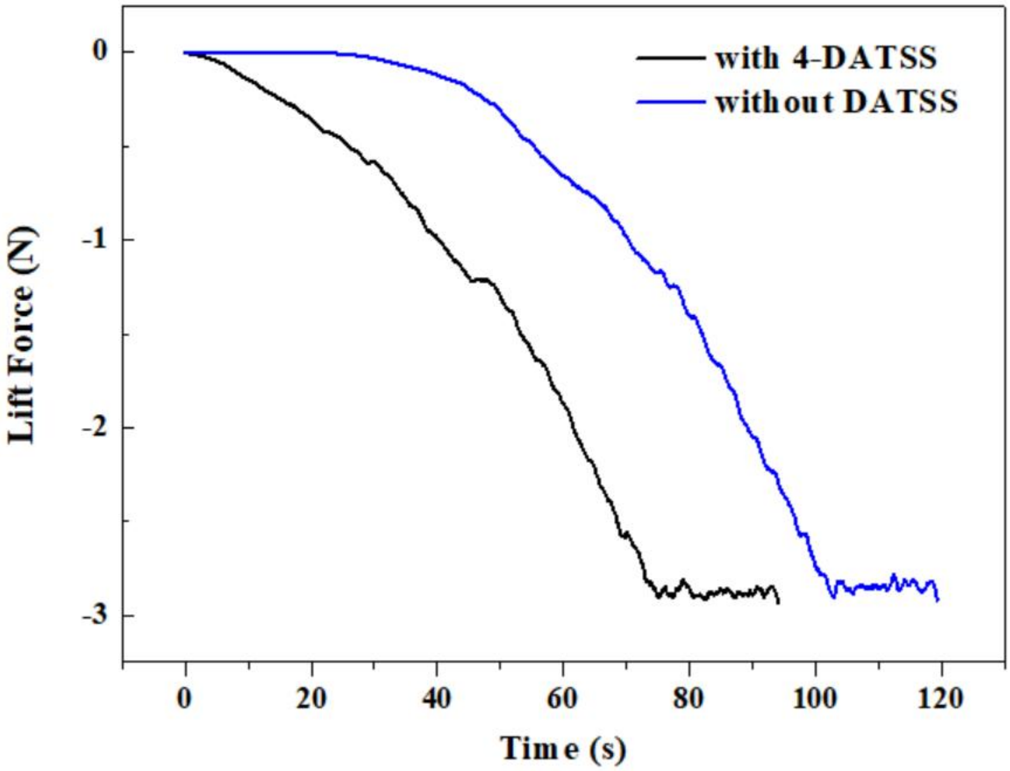

Supplementary Fig. 15 The airfoil lift with four DATSS units and without DATSS.

(4) CFD simulation. In addition to the analysis and discussion from the experimental point of view, we also considered the influence of the existence of the sheet on the lift and drag of the aircraft wing from the point of view of CFD simulation. Here, we consider the most extreme case, that is, to study how much lift and drag changes the steel sheet can bring to the airfoil without considering the aforementioned blocking backflow and downpressure airflow (both of which are helpful for lift). At the same time, in order to demonstrate the arrayable installation of the DATSS system, we placed four steel sheets on the wing section for simulation.

i) CFD parameter setting details: the incoming flow velocity is 200 m/s, the chord length is 0.3 m, and the span length is 0.5 m. Reynolds number: 4,107,522 (which can be identified as a high Reynolds number problem). Wing: NACA0012. Here we analyze the influence of the installation of the steel sheet on the lift drag of the airfoil. Since NACA0012 is a symmetrical wing, the lift of the symmetrical airfoil is basically close to 0 at an angle of attack (AoA) of 0°, which has no practical research significance. Therefore, we choose three cases where the AoA is 6°, 8°, 10° for CFD simulation. The software used is ANSYS2020-R2.

ii) Description of simulation mesh convergence: when there is no steel sheet on the airfoil, five convergence curves are calculated as residual, lift coefficient, drag coefficient, lift and drag curves as shown in Supplementary Fig. 16-20. The airfoil model without steel sheet has a total mesh number of 2 million.

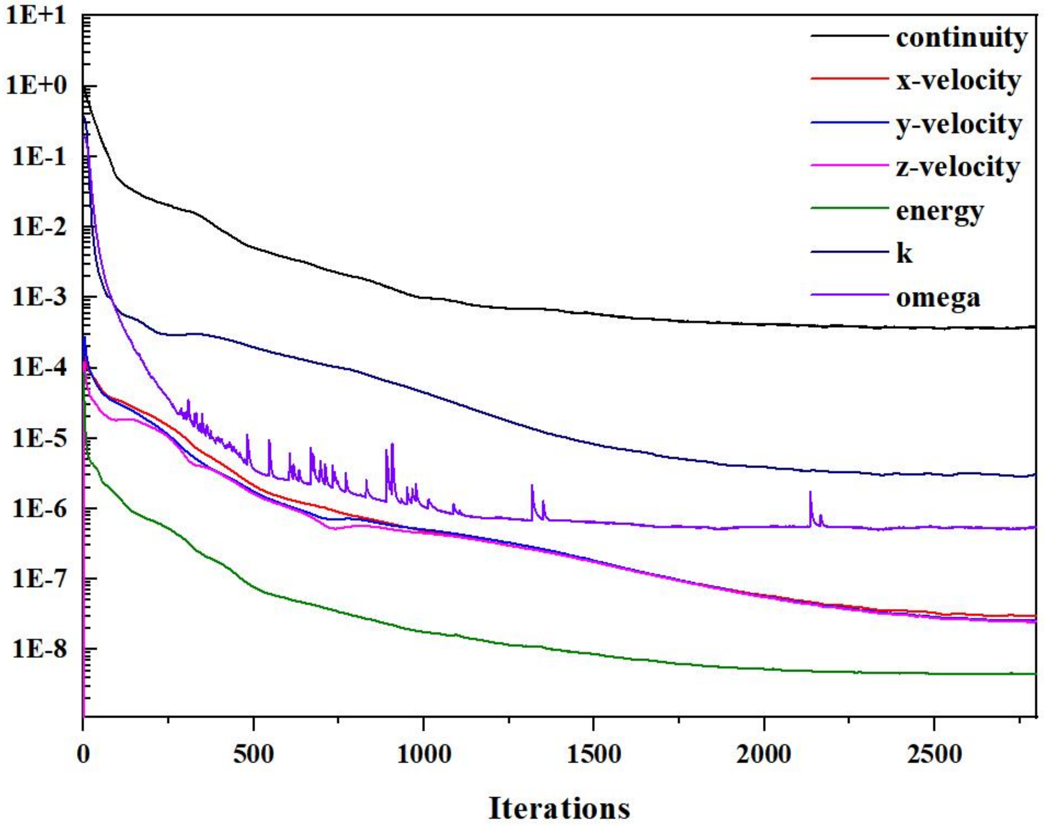

Supplementary Fig. 16 CFD simulation convergence calculation.

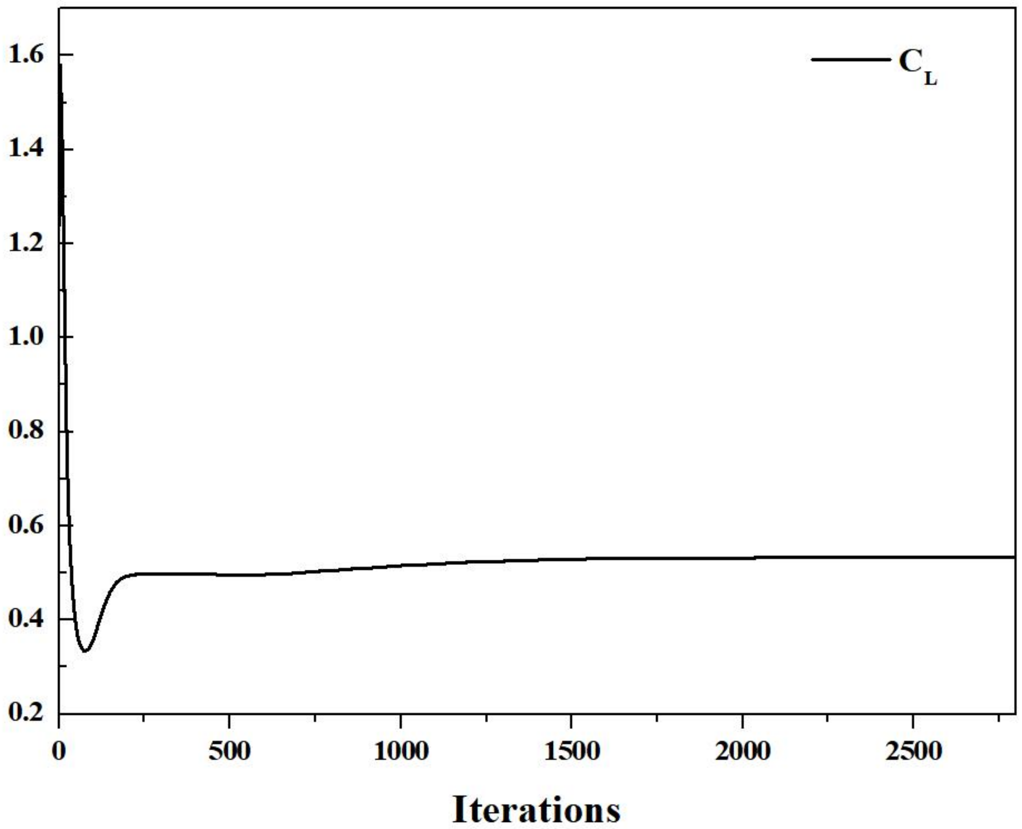

Supplementary Fig. 17 Lift coefficient curve without steel sheet.

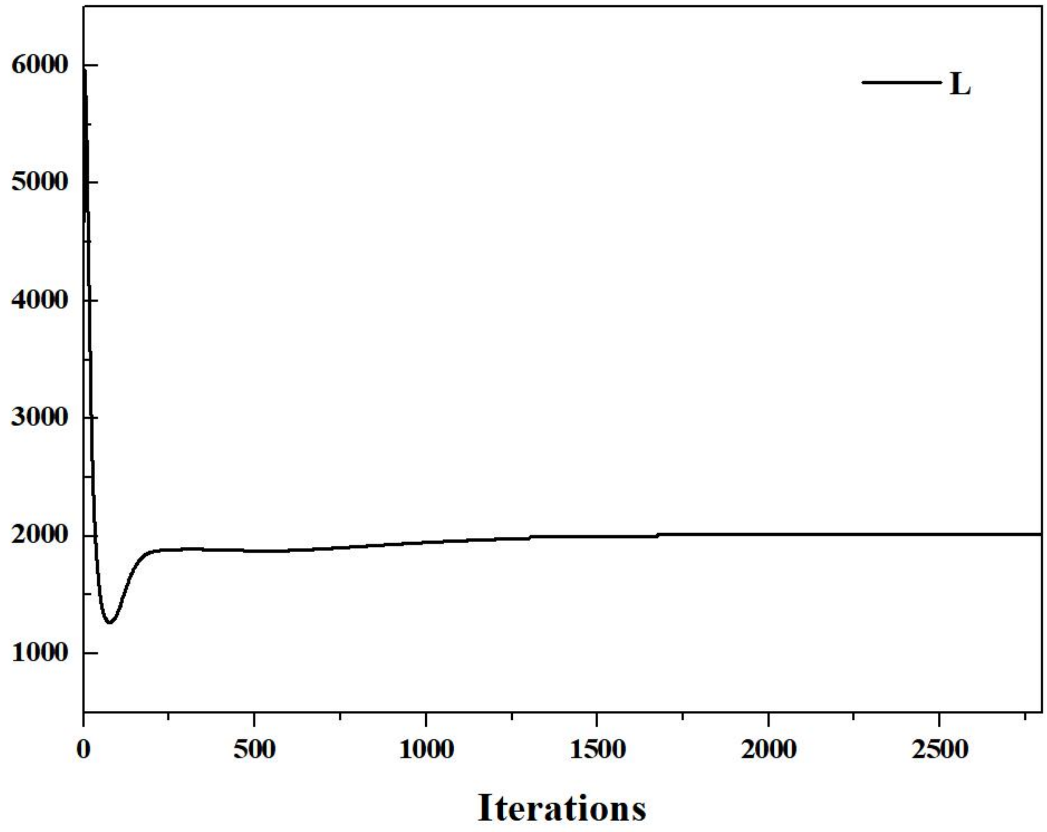

Supplementary Fig. 18 Lift curve without steel sheet.

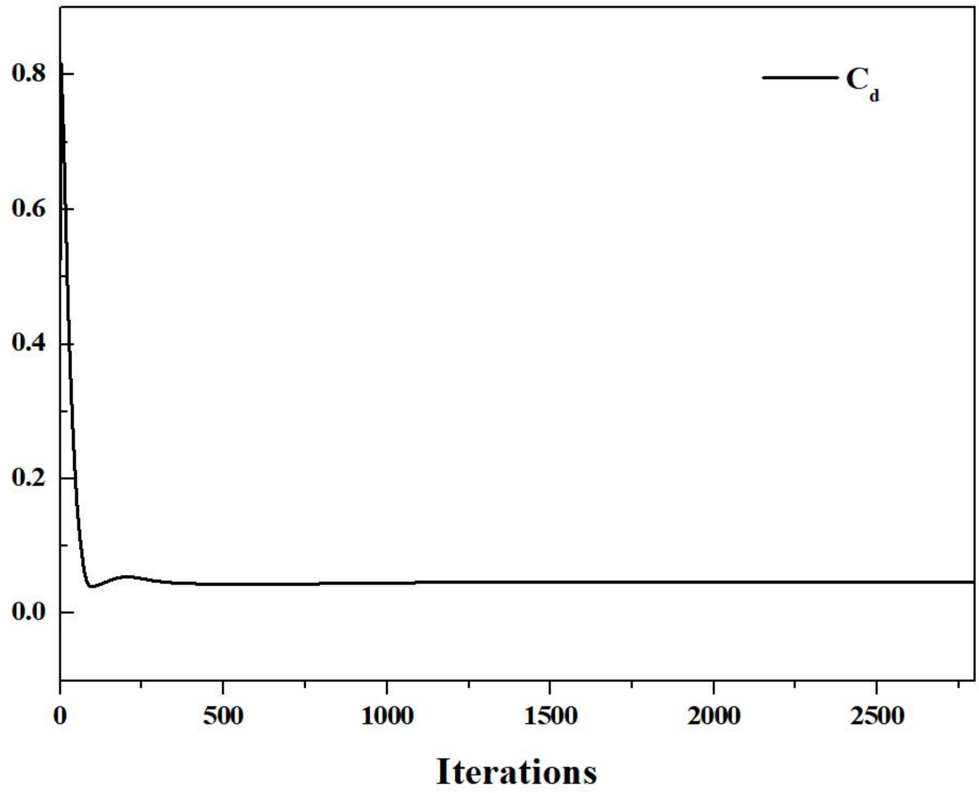

Supplementary Fig. 19 Drag coefficient curve without steel sheet.

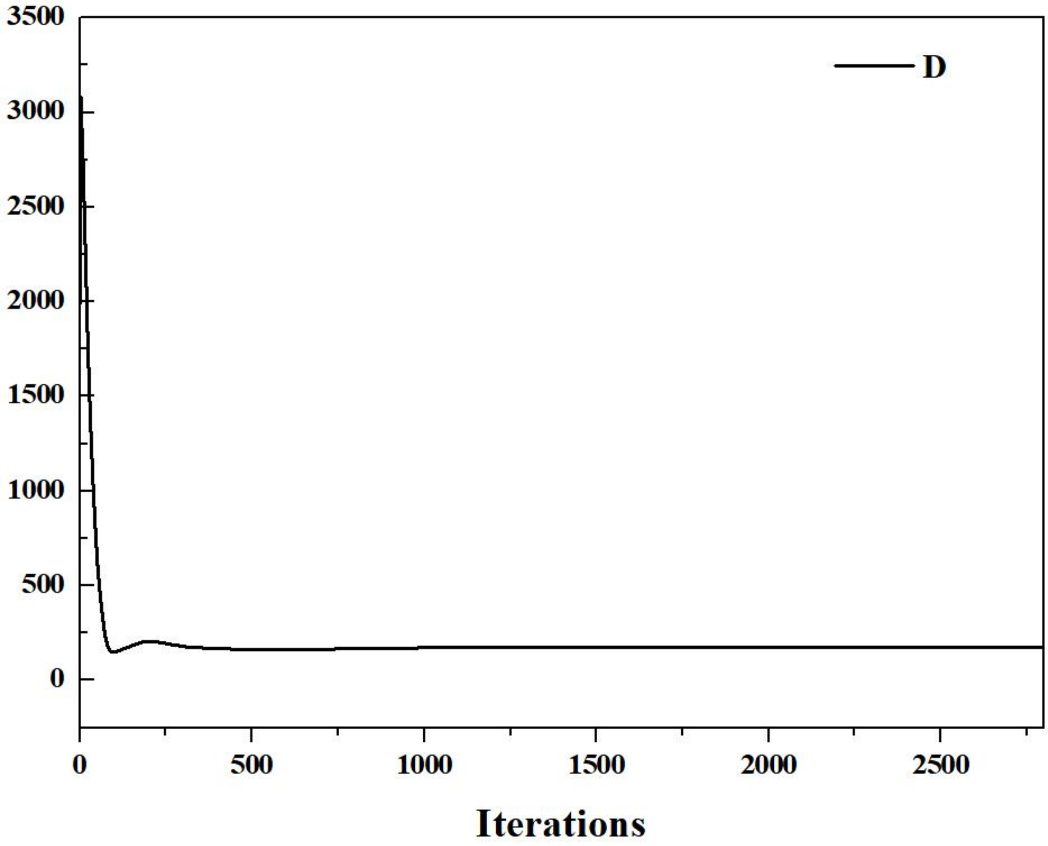

Supplementary Fig. 20 Drag curve without steel sheet.

iii) The convergence curves, lift coefficient, lift, drag coefficient and drag curves with steel sheet on the airfoil are shown in Supplementary Fig. 21-25 (because the number of meshes of the 3D model in this simulation is huge and the trial calculation time is long, here we only show the first 2800 steps of the curve of the trial calculation, and the trend can be seen from the curve, and the subsequent specific calculations have been completed on the supercomputer). The total number of grids of the airfoil model with steel sheets is 10.55 million, and the height of the first layer of grids is 0.002 mm.

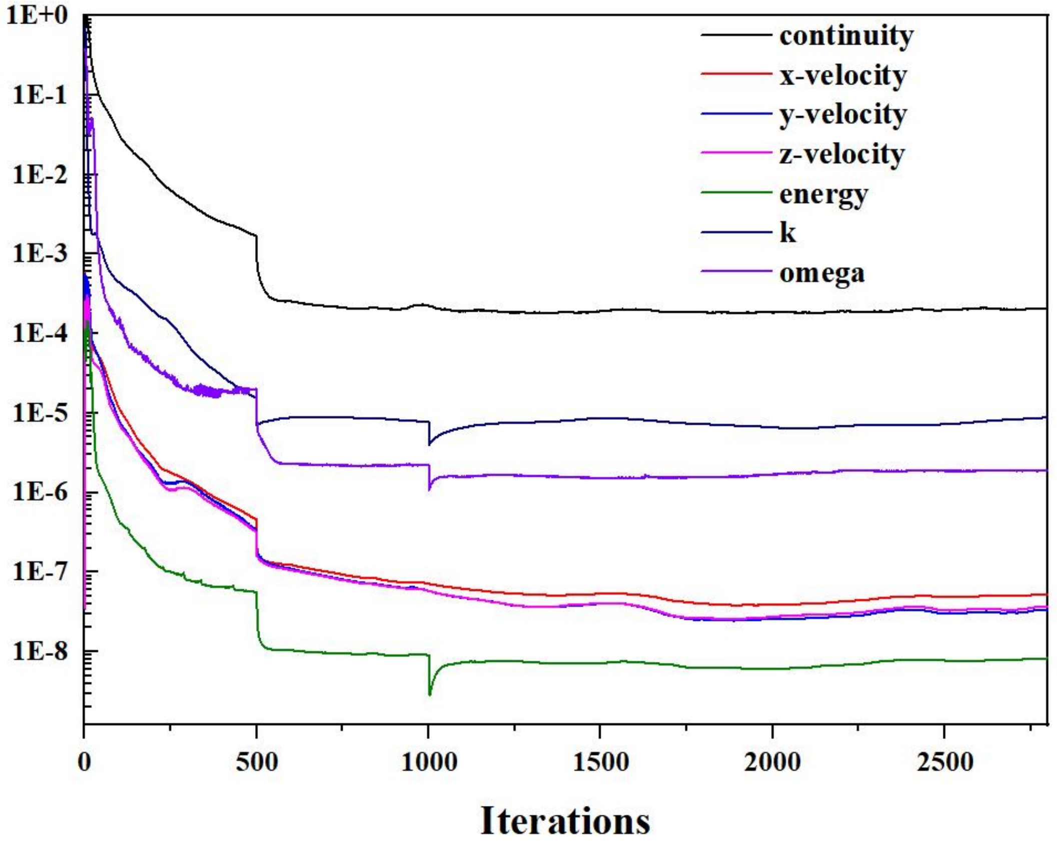

Supplementary Fig. 21 The convergence curve with steel sheet.

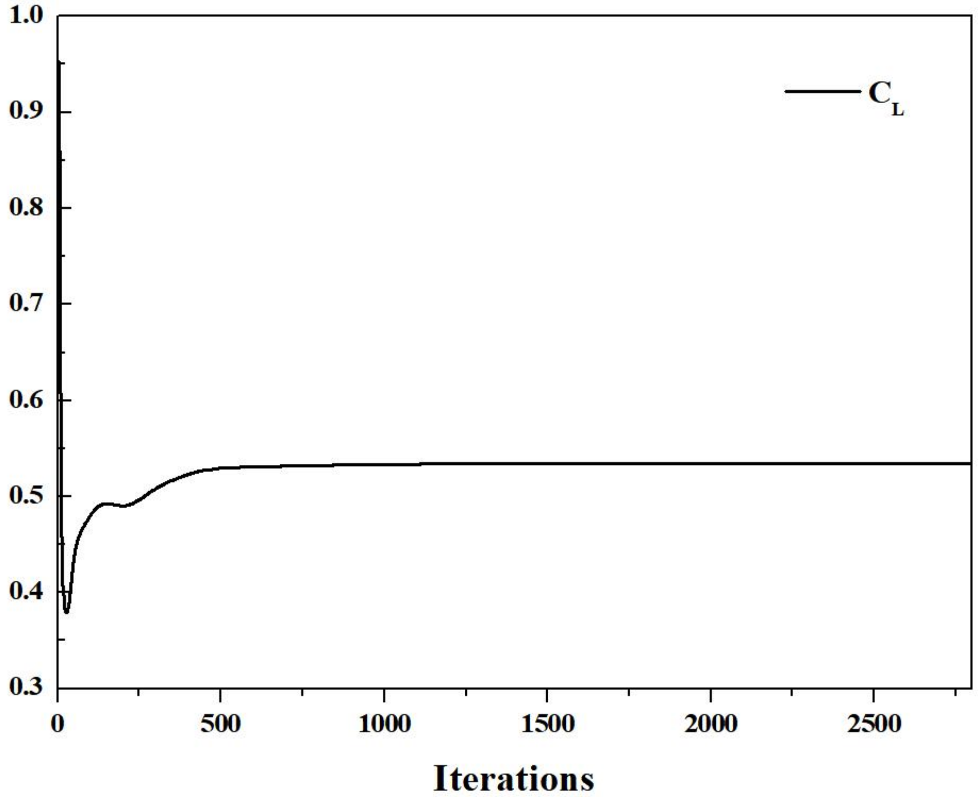

Supplementary Fig. 22 Lift coefficient curve with steel sheet.

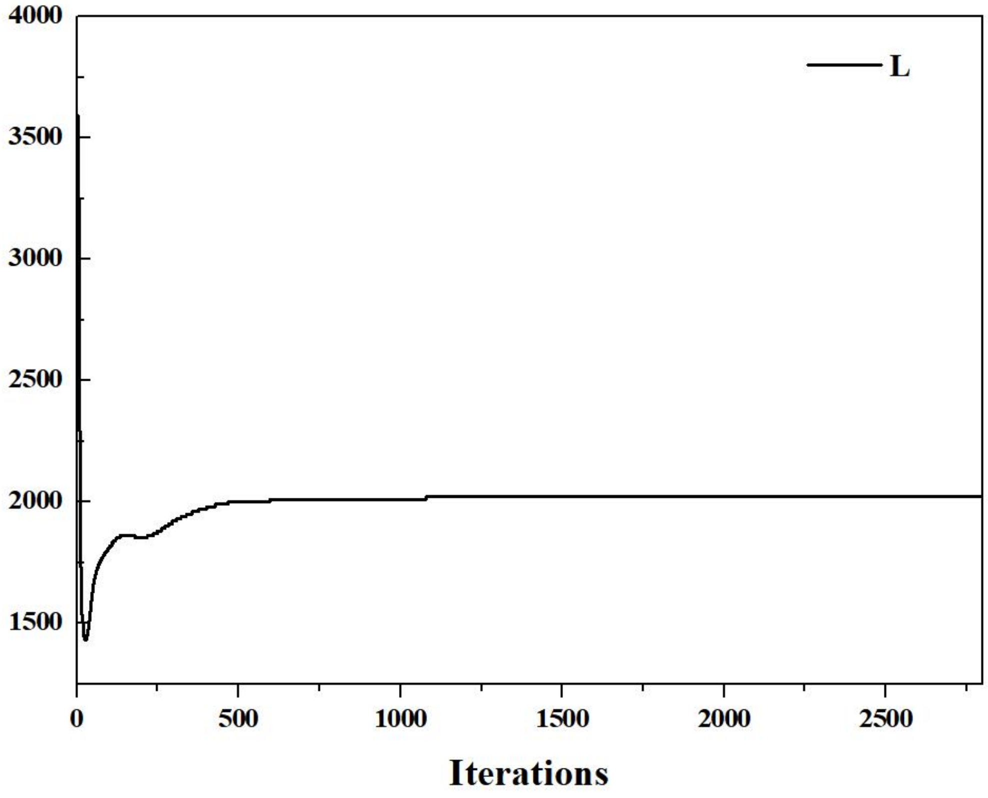

Supplementary Fig. 23 Lift curve with steel sheet.

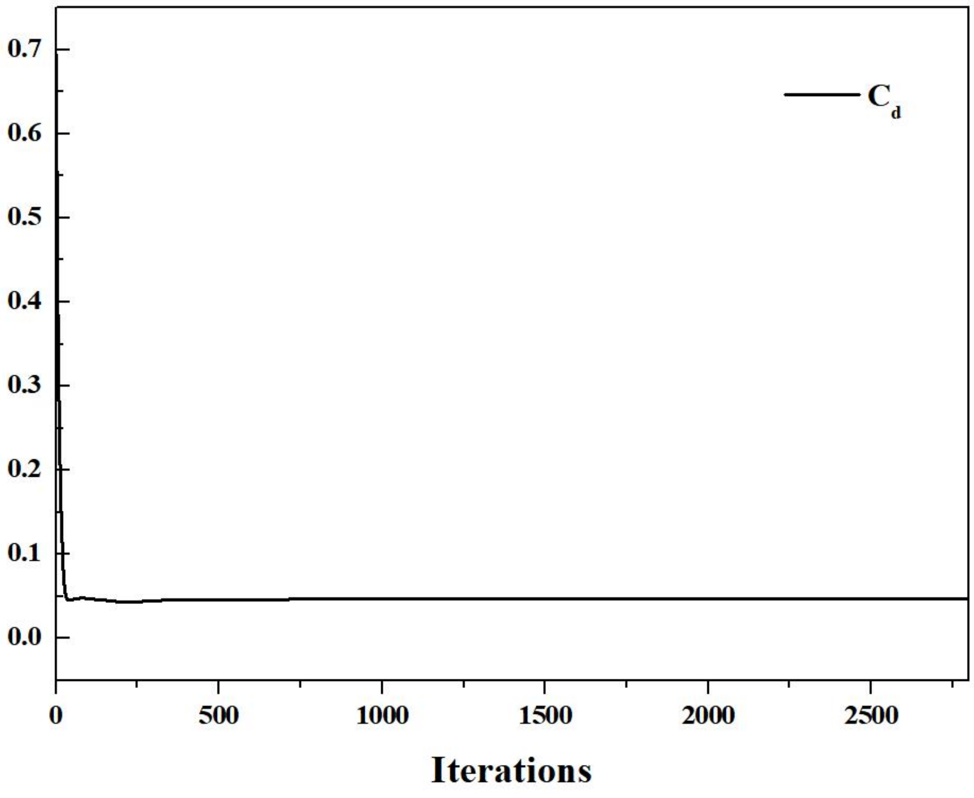

Supplementary Fig. 24 Drag coefficient curve with steel sheet.

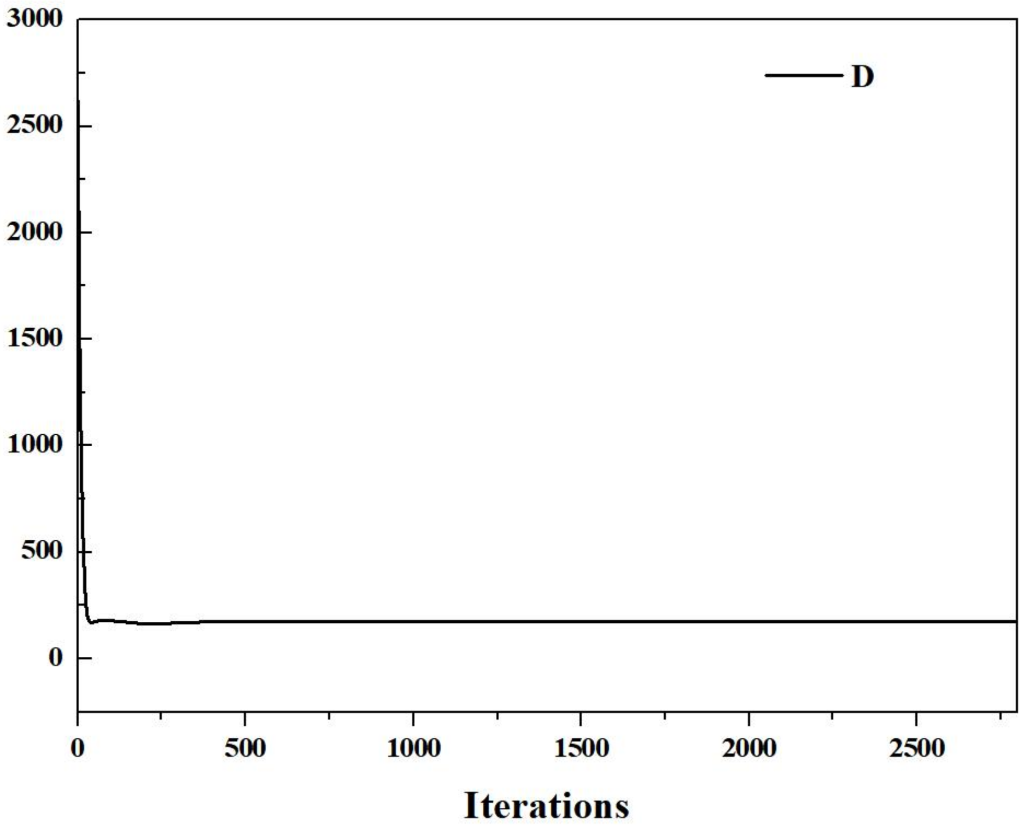

Supplementary Fig. 25 Drag curve with steel sheet.

iv) ICEM grid and fluent settings: the unstructured grid is used for division, and the size of the external flow field is 10 times the chord length. When meshing, the leading and trailing edges of the wing and the tip of the wing are locally refined. At the same time, the steel sheet needs to be encrypted for the model. Since the thickness of the steel sheet is ultra-thin and the distance to the upper airfoil is relatively close, the grid needs to be dense enough to ensure the restoration accuracy of the model. Boundary layers can also create problems that are difficult to generate because they are too close together. Therefore, we use the operation of making a boundary layer first, and then segmenting it to realize the generation of the boundary layer mesh. The grid diagram is shown in Supplementary Fig. 26-30. We can observe the position of the encrypted part and the boundary layer. According to the analysis of the calculation conditions, it is a high Reynolds number problem. It is roughly estimated that the grid height of the first layer is 0.002 mm, and there are 15 layers in total.

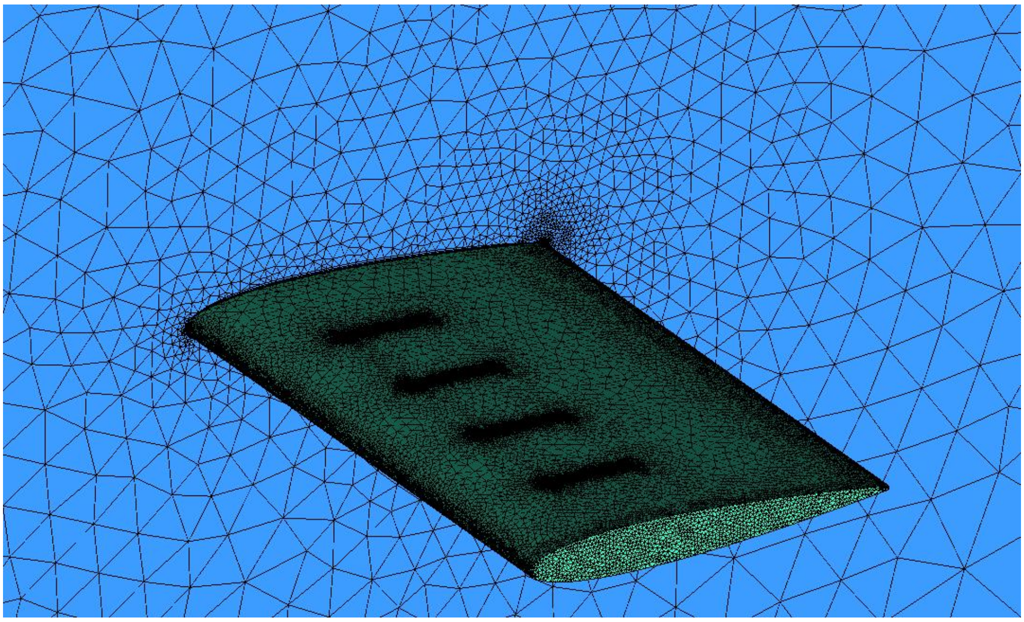

Supplementary Fig. 26 Schematic diagram of CFD model grid distribution.

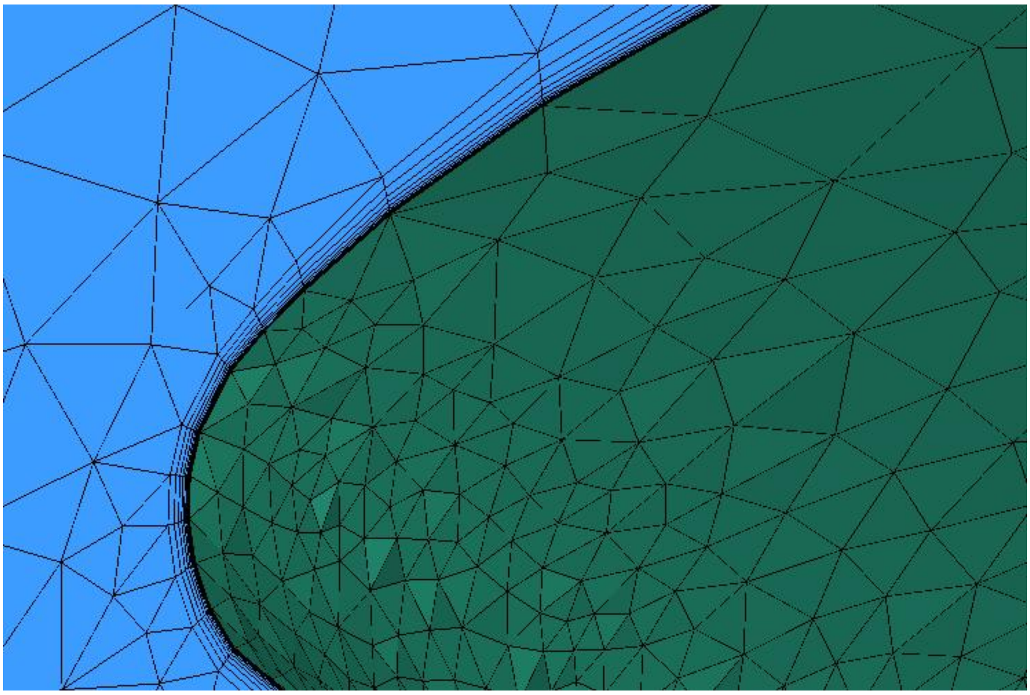

Supplementary Fig. 27 Schematic diagram of CFD wing front grid distribution.

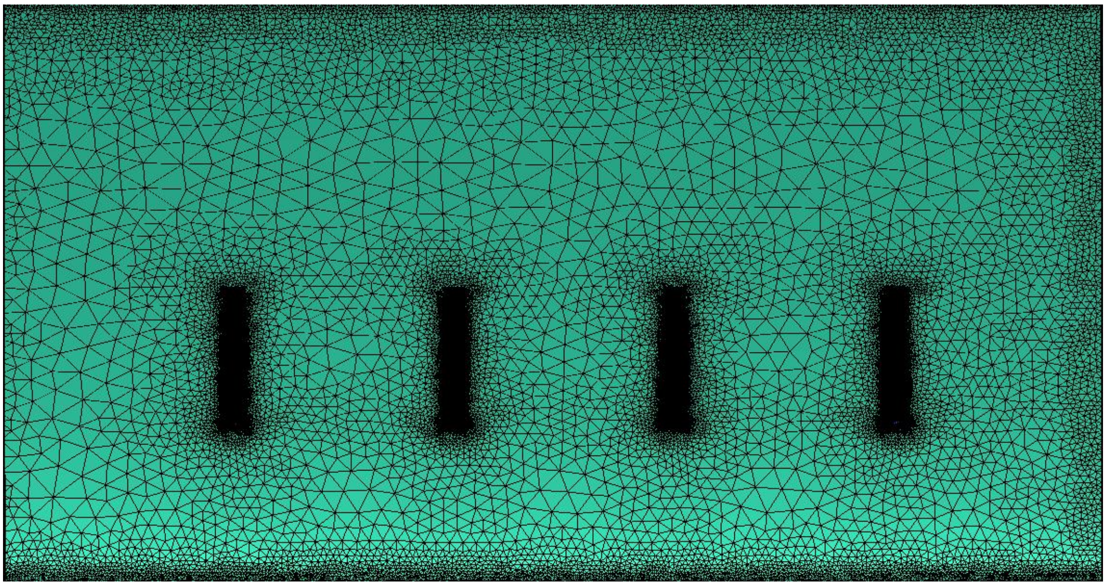

Supplementary Fig. 28 Schematic diagram of DATSS systems grid distribution.

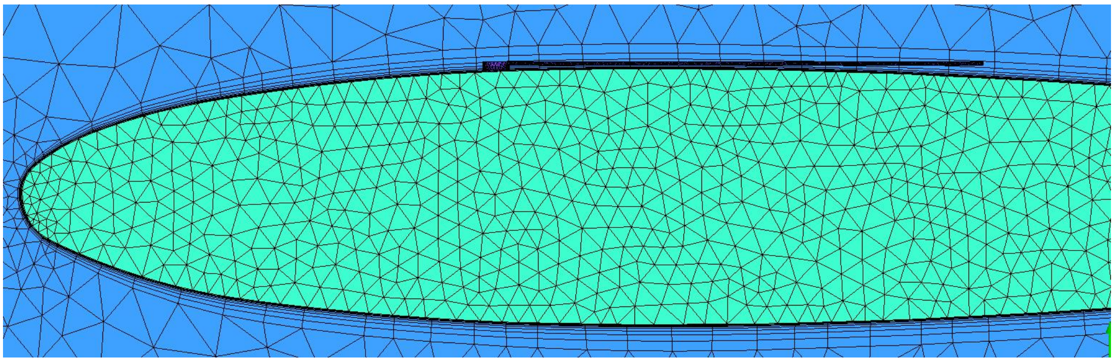

Supplementary Fig. 29 Side view of CFD modeling grid distribution.

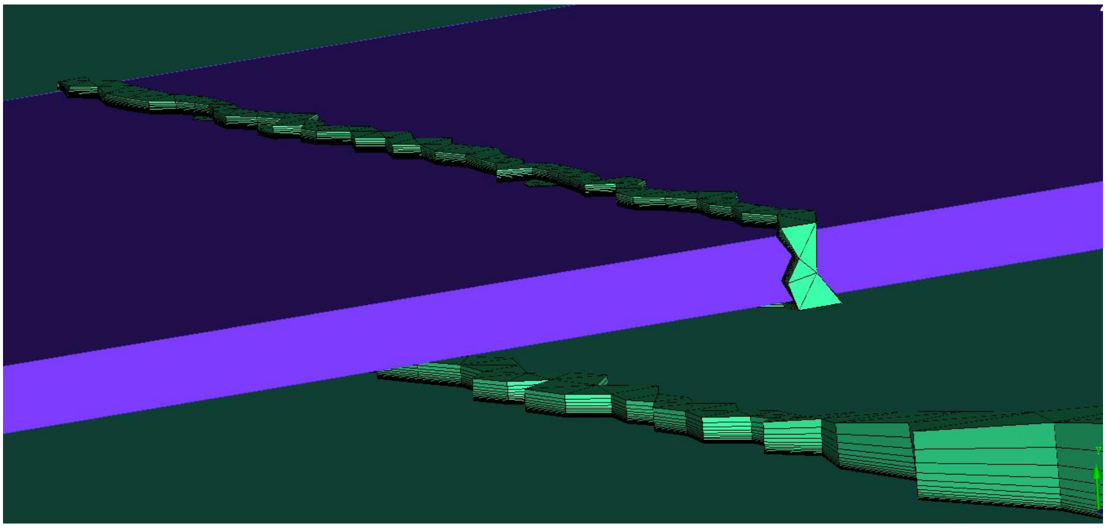

Supplementary Fig. 30 A local diagram of the DATSS system modeling subdivision grid.

v) Software parameter setting (ANSYS2020-R2):

No.	Set	Sort	Parameter
1	Solver	Type	Pressure-Based
		Velocity formulation	Absolute
		Time	Steady
2	Models	Energy	On
		Viscous	*SST-K-ω*
3	Materials	Air	Ideal-gas
		Viscosity	Sutherland
4	Operating Conditions	Operation Pressure	101,325 Pa
5	Boundary Conditions	Mach Number	0.5877270830
		X-Component of Flow Direction	6°, 8°, 10°
		Y-Component of Flow Direction	6°, 8°, 10°
		Turbulence Intensity	5%
		Turbulent Viscosity Ratio	10
6	Solution Methods	Scheme	SIMPLEC
		Skewness Correction	0
		Gradient	Least Squares Cell Based
		Pressure	Second Order
		Density	Second Order Upwind
		Momentum	Second Order Upwind
		Turbulent Kinetic Energy	First Order Upwind
		Specific Dissipation Rate	First Order Upwind

vi) Calculation results:

	No steel sheet installed				Steel sheet installed				lift coefficient (C_l) ratio	drag coefficient (C_d) ratio
AoA	C_{l-1}	L_1	C_{d-1}	D_1	C_{l-2}	L_2	C_{d-2}	D_2	C_{l-1}/C_{l-2}	C_{d-1}/C_{d-2}
6°	0.401656	1517.2147	0.031661	119.59552	0.401226	1515.5920	0.031389	118.5695	1.001071715	1.008665456
8°	0.533488	2015.19929	0.0467828	176.71777	0.533979	2017.0561	0.0465215	175.7056	0.999080488	1.005616758
10°	0.650746	2458.13043	0.0669403	252.860878	0.641714	2424.0139	0.0659274	249.0345	1.014074806	1.015363870

The unit of force is N. The convergence trend of small AoA is stable, and the monitoring data is basically stable. The maximum residual value of the final calculation is on the order of 1E-3, and the monitoring data changes very slowly,

which can be judged as convergent. Through the simulation analysis, it can be seen that the small enough sheet structure has little effect on the lift before the wing stalls, about 0.1%. If the width of the sheet is further reduced, the effect similar to that of a vortex generator can be achieved. By creating a vortex above the airfoil to accelerate the airflow velocity on the upper airfoil, the effect of increasing lift and reducing drag can be achieved. At the same time, we have analyzed in the preceding paragraphs that the downward pressure air effect of the steel sheet and the blocking backflow effect can have a positive impact on the lift force.

Supplementary Note 5

An in-depth exploration of the T/P-signal

In this part, we do an in-depth analysis of the T/P-signal. For the T-signal under different AoA and different wind speeds, we have done a more systematic study, as shown in Supplementary Fig. 31. After further study, we found that there is a relationship between the frequency of T-signal and the speed of wind tunnel motor (wind speed), in other words, in the DATSS system, we can also judge the current wind speed (flight speed) according to the T-signal, which makes the function of the DATSS system has been increased. At the same time, we also find that there are many reports on the correlation between the vibration frequency of T-signal and wind speed, but there are few reports under high wind speed. Therefore, this conclusion can supplement the signal characteristics of T-signal under high wind speed. But for the T-signal amplitude, the test uses a Keithley 6514 electrometer for a standard test, and its amplitude increases slightly with wind speed. In addition, we tested the frequency change of T-signal with AoA of 0° to 10° at the same wind speed. It was found that taking motor speed of 1500 rpm as an example, the frequency of T-signal was all around 40 Hz, and no correlation was found.

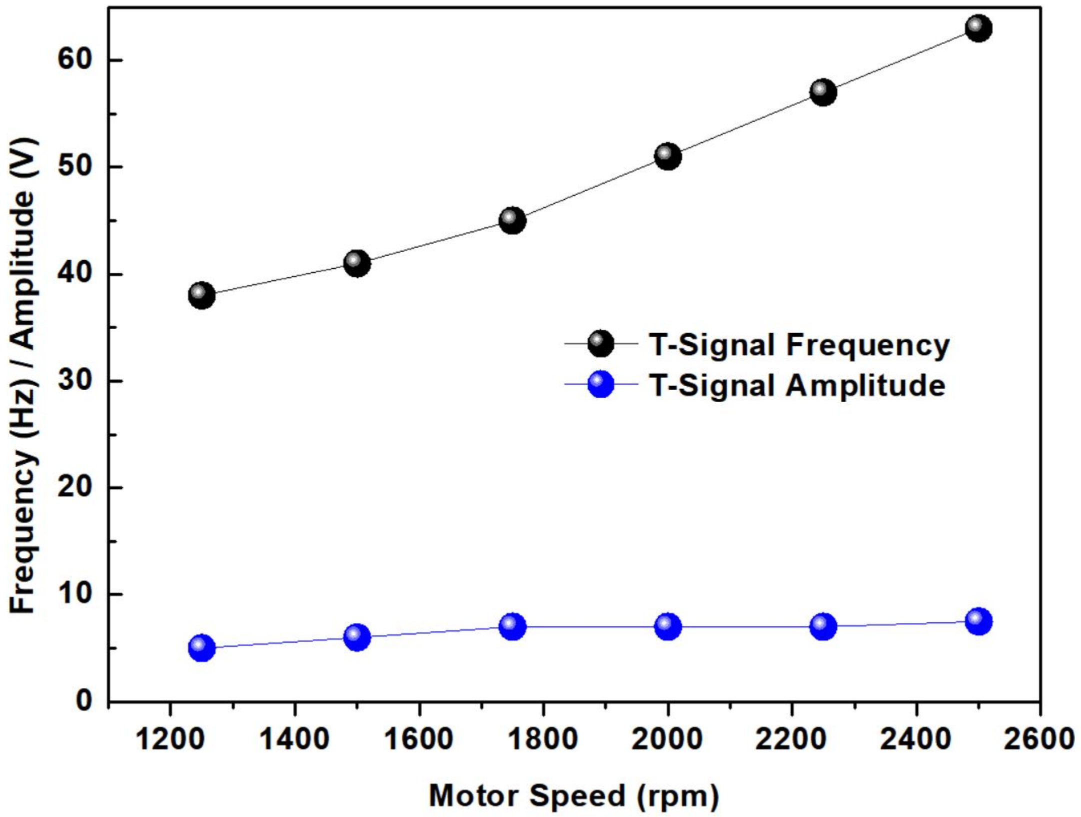

Supplementary Fig. 31 The relationship between T-signal frequency/amplitude and fan motor speed.

For the P-signal, we tested the sensitivity of the P-signal, since the piezoelectric effect is more of the effect of force on the voltage signal, the amplitude change of the P-signal at different bending angles is greater than the frequency change. First of all, we have carried out experimental research on the relationship between the rolling force and the angle of the steel sheet that generates the P-signal as shown in Supplementary Fig. 32.

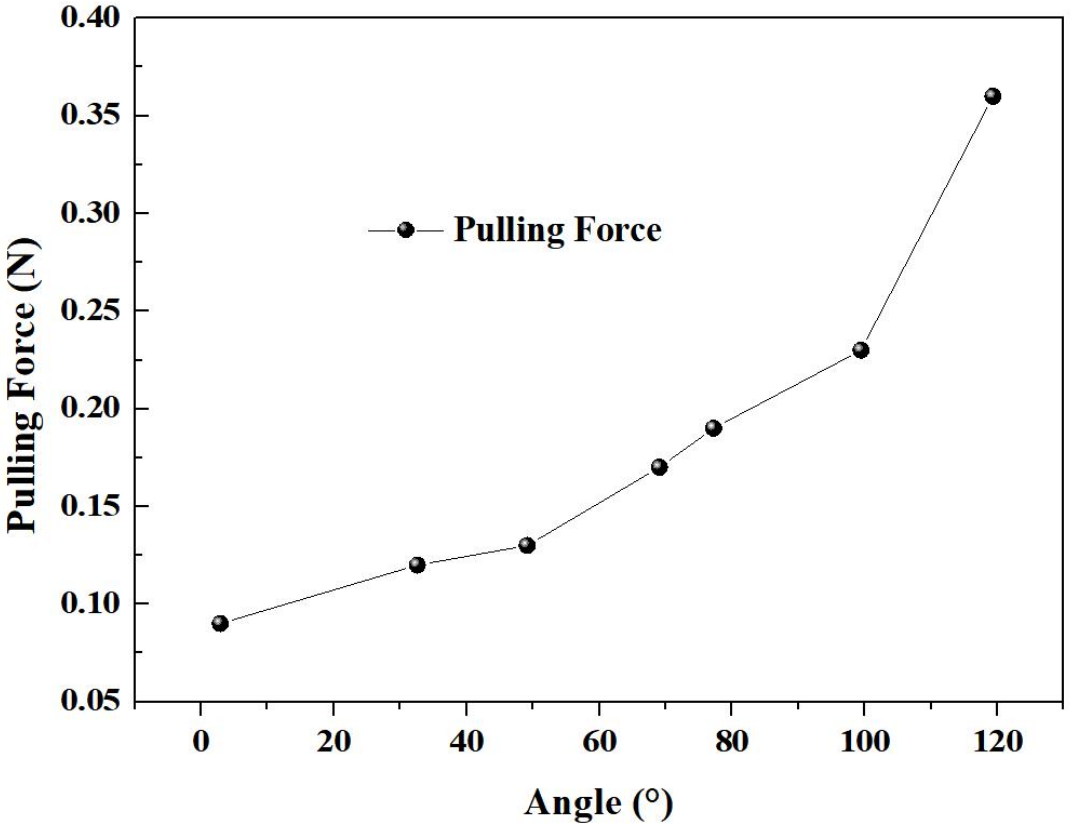

Supplementary Fig. 32 The relationship between the rolling force and the angle of the steel sheet.

It can be seen from the results that only 0.09 N of airflow separation force is needed to generate the P-signal, as the bending angle increases, the required force also increases. This is also the principle that the stall depth can be judged based on the P-signal amplitude. Related supplementary experimental pictures are as follows (Supplementary Fig. 33).

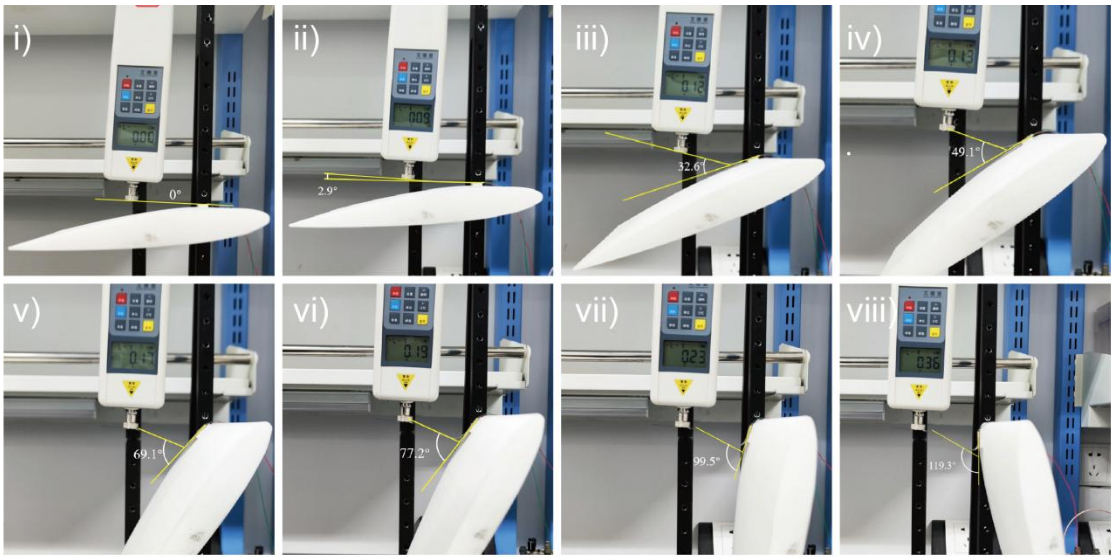

Supplementary Fig. 33 Photograph of the test of the relationship between the rolling force and the angle of the steel sheet.

At the same time, we measured the P-signal peak value under different bending angles several times, as shown in Supplementary Fig. 34. The stall depth can be roughly judged according to the amplitude of the P-signal. In future commercial products, the relative change of the P-signal amplitude may be used to analyze the degree of the stall, however, it is necessary to overcome the interference of signal in complex turbulent environment.

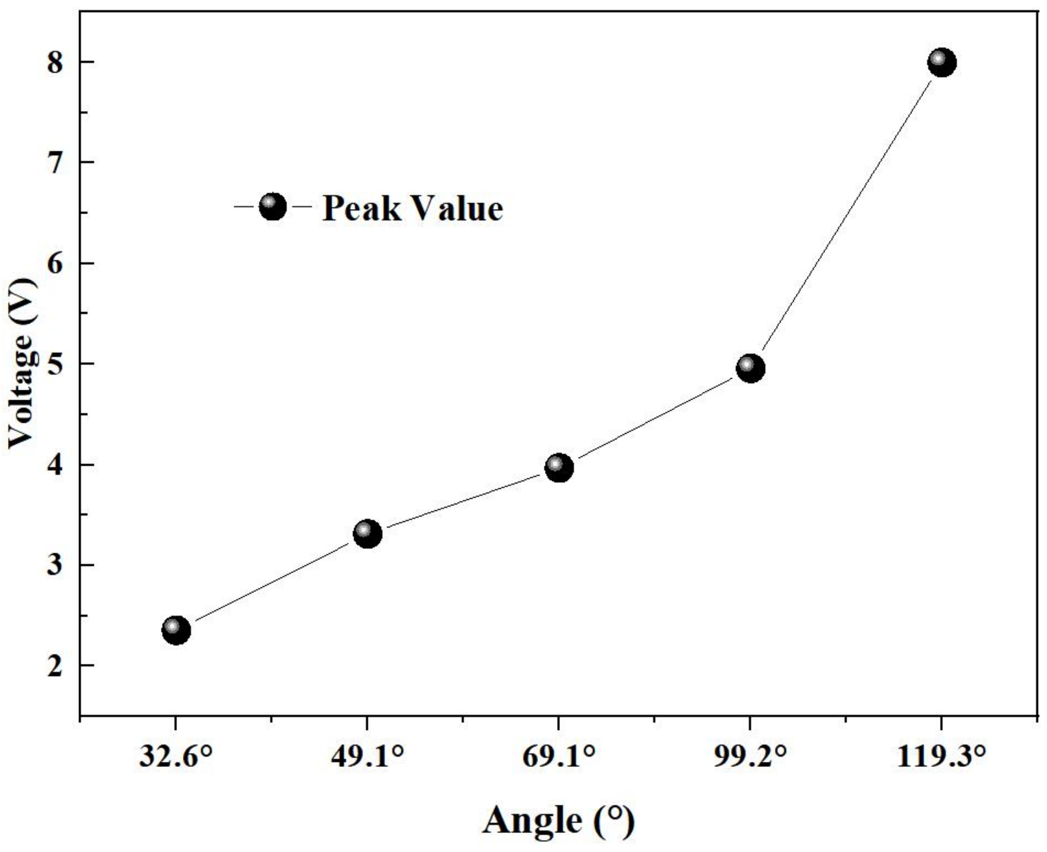

Supplementary Fig. 34 The P-signal peak value under different bending angles.

Finally, we also explored the effect of frequency variation on the amplitude of the P-signal, frequency has an effect on the amplitude of P-signal. Supplementary Fig. 35-37 are the results of measuring the relationship between frequency and amplitude of P-signal with mechanical motor at the bending angle of 30°, 60° and 90° respectively. We test the amplitude change of P-signal at the frequency from 1 Hz to 7 Hz when the bending angle is 30°. It is observed that the amplitude of P-signal tends to increase with the increase of frequency. However, after 4 Hz, this trend gradually slows down and becomes stable. Since the actual operating frequency of the DATSS system is greater than 20 Hz, in summary, the influence of the bending angle on the amplitude of P-signal is greater than that of the frequency.

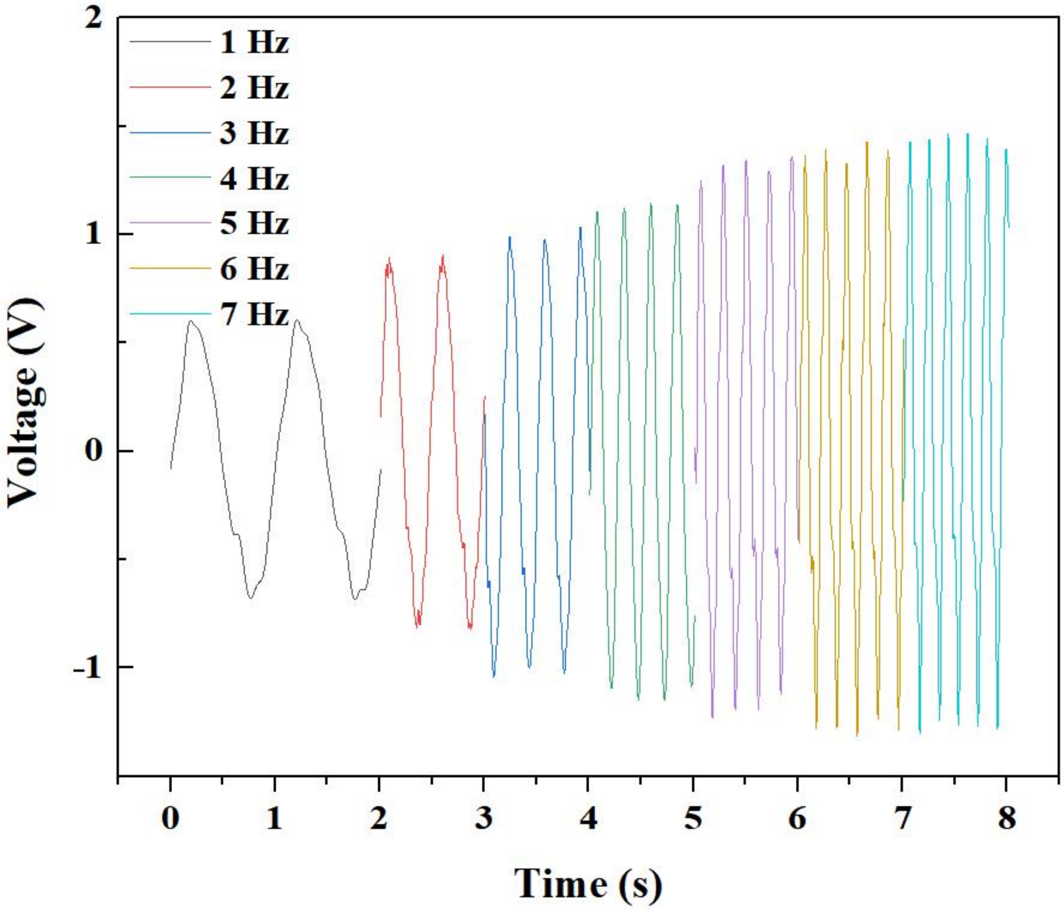

Supplementary Fig. 35 The relationship between frequency and amplitude of P-signal with mechanical motor at the bending angle of 30°.

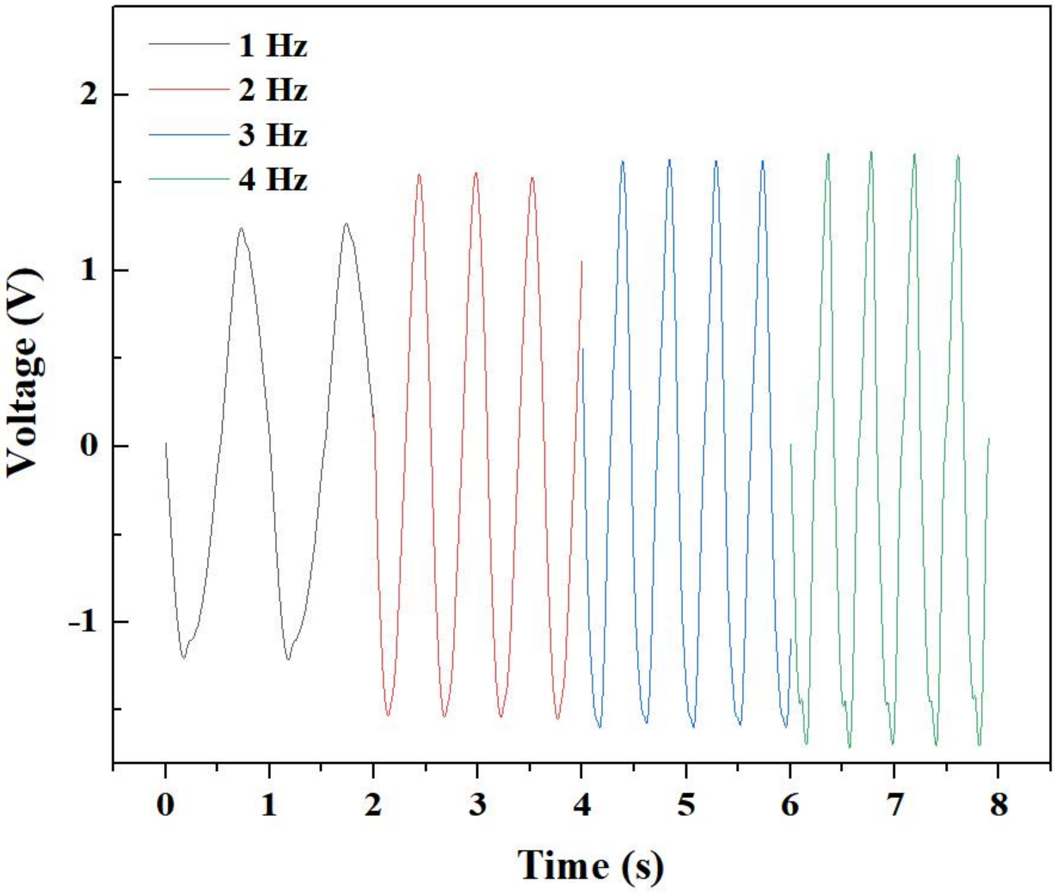

Supplementary Fig. 36 The relationship between frequency and amplitude of P-signal with mechanical motor at the bending angle of 60°.

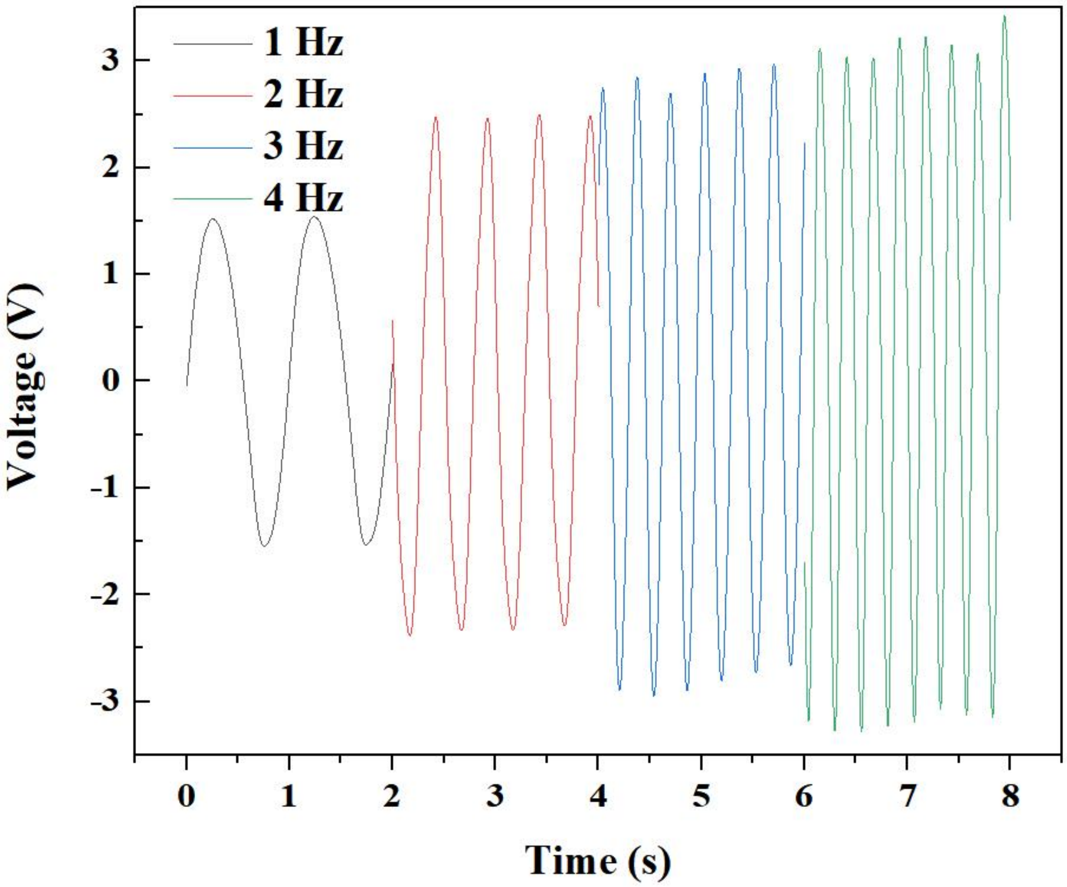

Supplementary Fig. 37 The relationship between frequency and amplitude of P-signal with mechanical motor at the bending angle of 90°.

Supplementary Note 6

Large Reynolds number experiments and limitations of DATSS systems

In this part, we discuss the possible shortcomings of DATSS system for high Reynolds number wind tunnel experiments. In order to achieve the real Reynolds number level, we made a wing with a large chord length (600 mm), a width of 422.60 mm. The AoA can be changed through a circular rotating shaft in the middle of airfoil as shown in the figure. T/P-signal is the whole process signal when the AoA changes from 0 degrees to 16 degrees and then to 0 degrees. At the same time increased the flow velocity of the wind tunnel (80 m/s), keeping the room temperature the same as the outdoor (0 ℃, increasing the air density), and finally made the Reynolds number

reach 3×10^6, which is the Reynolds number of real aircraft such as Cessna 172, and is smaller than the Reynolds number of large aircraft such as Boeing 7-series aircraft. Under the DATSS system with the same design, the test results are shown in Supplementary Fig. 38 (using the same test single chip microcomputer as the flight in the field). We showed the original data of the signal, from which we can analyze: i) the amplitude of T-signal increases after the Reynolds number increases. ii) As the Reynolds number increases, the noise signal increases. iii) P-signal increases slightly after Reynolds number increases. iv) It can be seen from the signal shape that the real Reynolds number airflow field model changes little, which is consistent with the speculation of self-similarity region. At the same time, it is worth noting that the signal trend under the real Reynolds number in the wind tunnel is the same as that under the field flight, but different in amplitude.

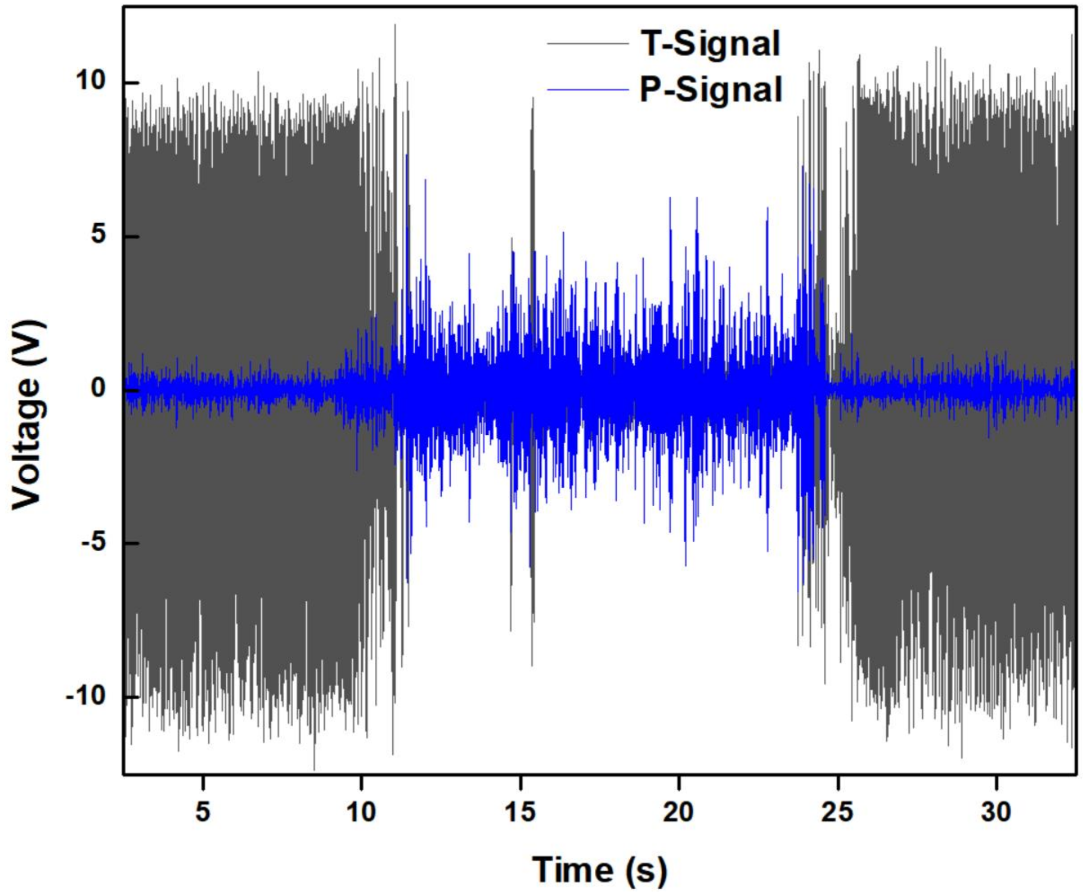

Supplementary Fig. 38 The T/P-signal test of DATSS system at the Reynolds number reach 3×10^6.

For the limitations of the DATSS system, since the vibration and rollover of the steel sheet need to be stressed, the minimum force to support its vibration and rollover is the limit of the working conditions of the DATSS system. After experiments, we found that when the incoming flow velocity in the wind tunnel is less than 15 m/s, no matter how the AoA changes, the T/P-signal cannot be generated. Therefore, the working speed greater than 15 m/s is the limitation of the DATSS system for the minimum working speed (in general, civil aviation aircraft and most drones can meet this speed requirement).

Supplementary Note 7

Discussion the accuracy of DATSS system

First, we discuss the accuracy from the principle of the DATSS system, the principle of the stall warning of DATSS is *in situ* monitoring of the separation of airflow, which is essentially different from the AoA sensor. AoA sensors are indirectly monitored for stalls, when AoA is greater than the preset warning value, it will send out a warning to the pilot or drone controller, which may be cause a stall. (It is worth noting that the logic we are talking about here is that the aircraft may be stall because the AoA sensor cannot directly accurately judge the stall.) Stall is connected with different flight speeds, different aircraft wing parameters, and different flight environments. At this time, the AoA sensor can only play a warning role. Whether the stall is occurred requires the operator to judge independently. Therefore, based on the above facts, the DATSS system uses the airflow separation to determine the occurrence and depth of the stall, in principle, it is better than the AoA sensor.

Next, we discuss the accuracy of early warning of the DATSS system. Before answering this question, and at the same time before we design the DATSS system, we have already discussed the stall warning logic of the DATSS system, which stage of the stall needs to be warned, or when does the warning start? We know that it is inaccurate to simply monitor the aircraft AoA to warn of the stall. As the Reynolds number increases, the stall AoA will also increase (this increase is small), so the

DATSS system chooses to use airflow separation monitoring to warn of a stall. The broad definition of stall AoA is the AoA when the lift coefficient of the aircraft begins to decrease. As the AoA continues to increase, the lift coefficient decreases at an accelerated rate, eventually leading to a crash. We first calculated and simulated the lift coefficient curve of our wind tunnel experiment airfoil NACA0012, as shown in Supplementary Fig. 39. When the AoA is about 16°, the lift coefficient begins to decrease (the experimental simulation Reynolds number is about 600,000). For different software and different references, the calculation method of the lift coefficient is slightly different, so it is necessary to slightly adjust the early warning angle according to the measurement when it is commercially available. We tested the angle of attack of the DATSS system at P-signal generation 100 times, and the results all fell around 16.2 degrees. Since its principle is based on the sensing of airflow separation, its accuracy is higher than that of the AoA stall sensing scheme. In addition, we also calculated the drag coefficient and lift-to-drag ratio changes of the NACA0012 airfoil under different AoAs and different Reynolds numbers, which can be used as an experimental reference, as shown in Supplementary Fig. 40-41.

Finally we discuss the issue of Reynolds number. In wind tunnel experiments, when the Reynolds number is large, the turbulent eddy viscosity dominates, and the molecular viscosity is almost negligible. Therefore, as long as the Reynolds number is larger than a certain value, the flow is similar by default. This value is the value of the fully developed turbulent flow, which is generally considered to be 10^5-10^7. Therefore, as long as the Reynolds numbers of the real flow and the model experiment are large enough, they are considered to be similar, and this Reynolds number range is usually called the self similarity region. Therefore, we have set the Reynolds number to 10^5 in the design phase of the wind tunnel experiment. At this time, the boundary layer can be regarded as full of turbulent flow, and the influence of the Reynolds number (10^5-10^7) on the flow can be ignored. In this Reynolds number range, when other conditions remain unchanged, the airflow separation point basically remains

unchanged. Most commercial aircraft have a Reynolds number of 10^6, so our model is in a similar range to the real aircraft Reynolds number.

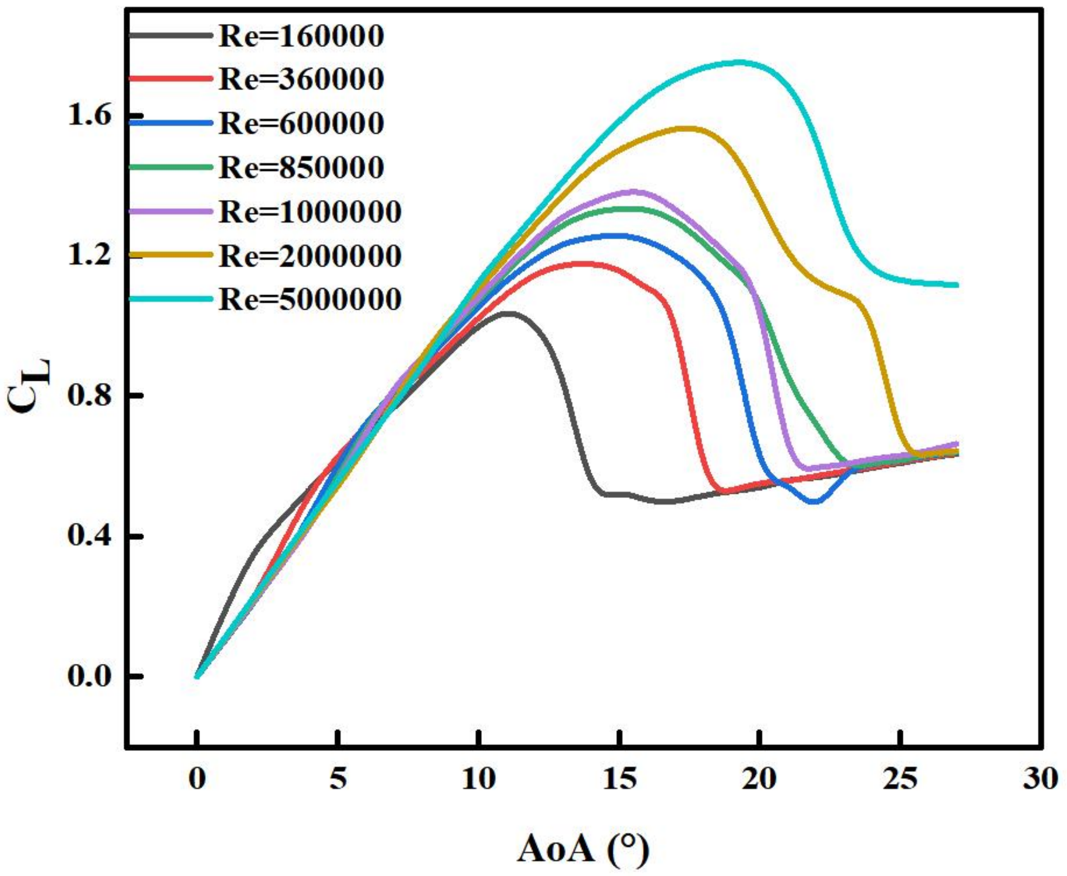

Supplementary Fig. 39 The lift coefficient curve of experiment airfoil NACA0012.

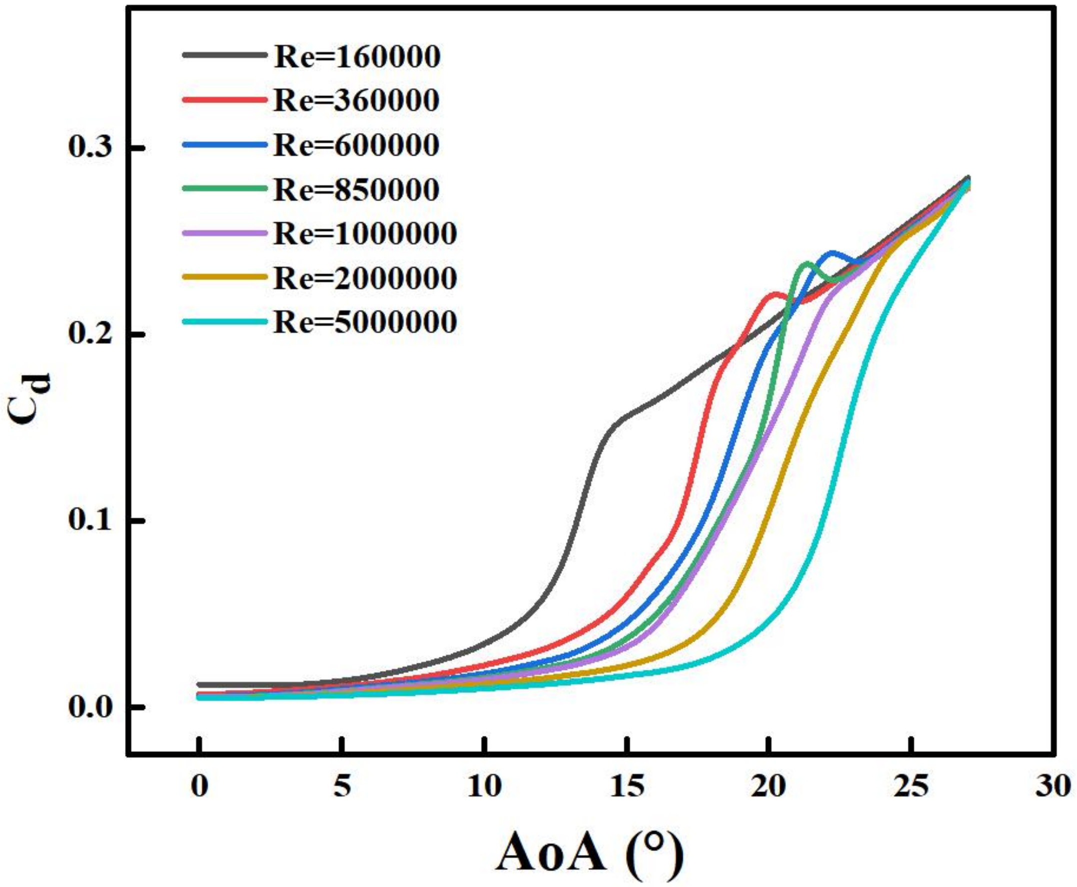

Supplementary Fig. 40 The drag coefficient curve of experiment airfoil NACA0012.

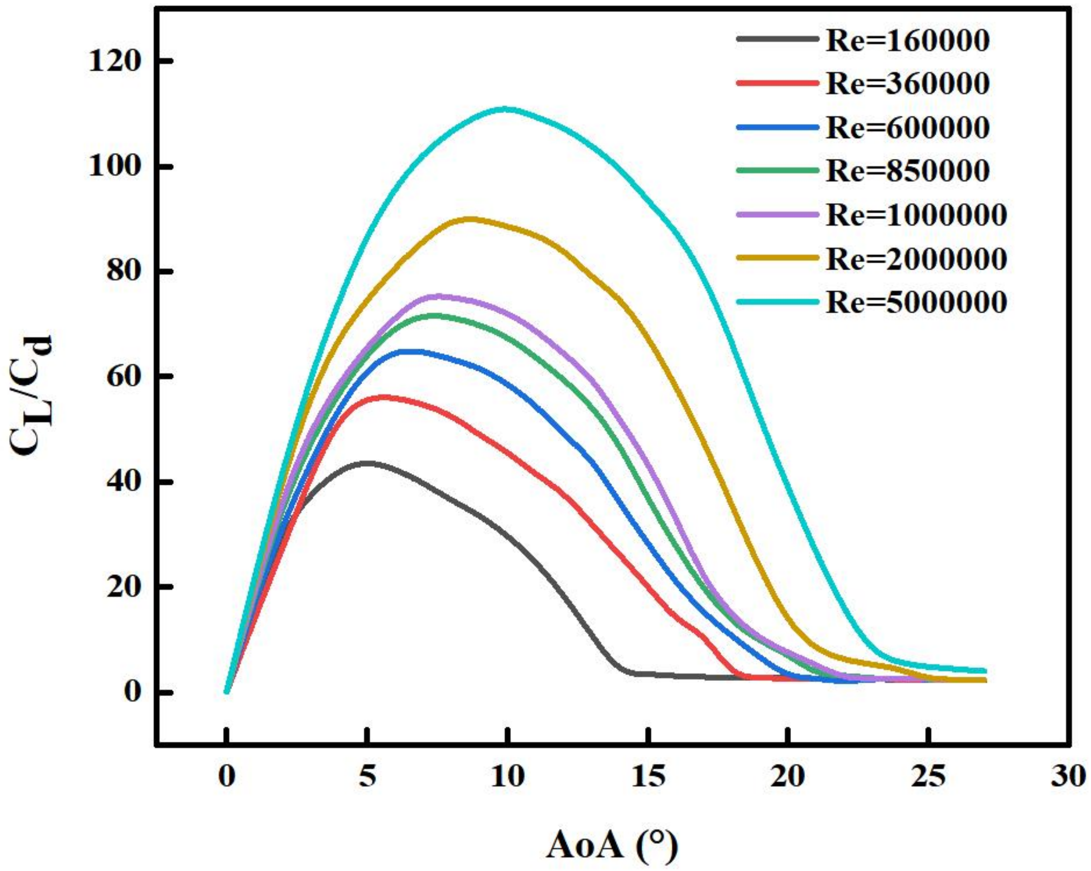

Supplementary Fig. 41 The lift-to-drag ratio changes curve of experiment airfoil NACA0012.

Here, it is worth noting that after answering the accuracy question, we would like to share the "customizable" features of the DATSS system as shown in Supplementary Fig. 42. The customization of the DATSS system is reflected in three aspects:

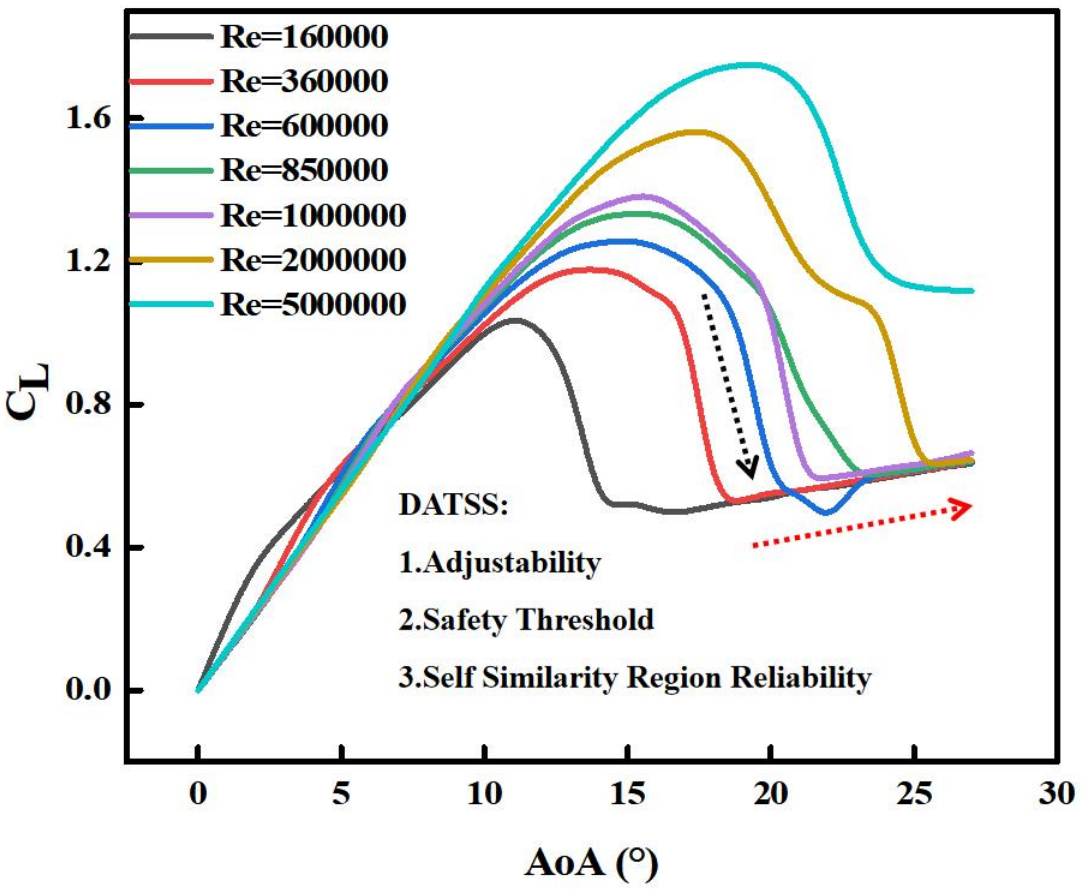

Supplementary Fig. 42 The lift coefficient curve of NACA0012.

(1) Adjustability. As shown by the black arrow in Supplementary Fig. 42, firstly, the stall is not a flight state corresponding to a specific AoA, and the stall is a whole range after the lift coefficient drops until the lift is lost. Different from existing AoA stall sensors, it can only warn the stall after a specific AoA. The early warning of the DATSS system can be artificially set at most locations in the entire stall area, which is in line with the early warning logic (it is not difficult to understand, only need to adjust the stiffness, thickness and length of the steel sheet, early warning can be given at the position where warning is needed). What are the benefits of this? The logic is that some pilots or UAV operators prefer to receive an early warning signal when the airflow separation begins, so as to ensure the flight safety of the aircraft to the greatest extent; on the contrary, other controller prefer the joy of controlling the aircraft with a large AoA, hoping to receive a stall warning when the airflow separation is serious. In

this way, the DATSS system with adjustable warning position is undoubtedly more advantageous.

(2) Safety Threshold. As shown by the red arrow in Supplementary Fig. 42, as the Reynolds number (Re) increases, the critical stall AoA of the aircraft will also increase. Therefore, in the wind tunnel test, we use a Reynolds number on the order of 10^5 (when Re>10^5, the wind tunnel can simulate the flow field with Re between 10^5 and 10^7 as the fluid model is similar) to ensure the safety critical threshold of the DATSS system. That is to say, at a small Reynolds number, the DATSS system can work normally in early warning, as the Reynolds number increases, the critical stall AoA of aircraft will only become larger, and no less than a low Reynolds number.

(3) Self Similarity Region Reliability. As mentioned in the answer to the real Reynolds number problem, in this work, it is not necessary to consider the influence of Mach number in low speed incompressible flow, and the flow state is mainly affected by Reynolds number. The various properties of incompressible flows are generally described as varying with the Reynolds number, and as long as the Reynolds number is approximate equivalence, it is usually ensured that the conclusions of model experiments can be applied to real flows. If the real flow field is too small to be measured (for example, the lift force of insect wings), a larger model can be used to replace it in the experiment, and the velocity can be reduced appropriately to ensure the approximate equivalence of Reynolds number. If, on the other hand, the actual flow field is too large to be achieved in a wind tunnel (such as studying the aerodynamic forces on a skyscraper), the size needs to be reduced and the velocity increased. In addition, many natural air flows are often tested in water tunnels, where the Reynolds number is approximate equivalence and the effect of gravity is excluded. This idea is fine in theory, but difficult to implement. For example, a skyscraper with a height of 400 m is limited by the size of the wind tunnel. If the model with a height of 0.4 m is used instead in the experiment, the wind speed in the wind tunnel needs to be 1000 times of the actual wind speed in order to ensure the same Reynolds number. If the actual wind speed is 10 m/s, then the wind speed in

the wind tunnel should be 10 km/s. Regardless of whether such wind speeds can be achieved in a wind tunnel, the point is that this is already hypersonic flow, and compressibility and changes in the physical properties of the air make the flow completely different. Therefore, even for incompressible flows, the Reynolds number of the model experiment is often not equal to that of the actual flow. How do we make sure that the flow is similar (approximate equivalence)? When the Reynolds number is large, the turbulence vortex viscosity takes the dominant position and the molecular viscosity can be almost ignored. Therefore, the flow is almost similar as long as the Reynolds number is large to a certain value, which is the value that guarantees the flow to be fully developed turbulence, generally believed to be $10^5 \sim 10^7$. Therefore, as long as the Reynolds numbers of the real flow and the model experiment are large enough, they are considered to be similar, and this range of Reynolds numbers is often referred to as the self-similarity region. Numerous wind tunnel experiments are also based on this. The airflow separation flow field we focus on in this work can be regarded as equivalent to the real flow field.

Supplementary Note 8

2D Working aerodynamic simulation of DATSS system

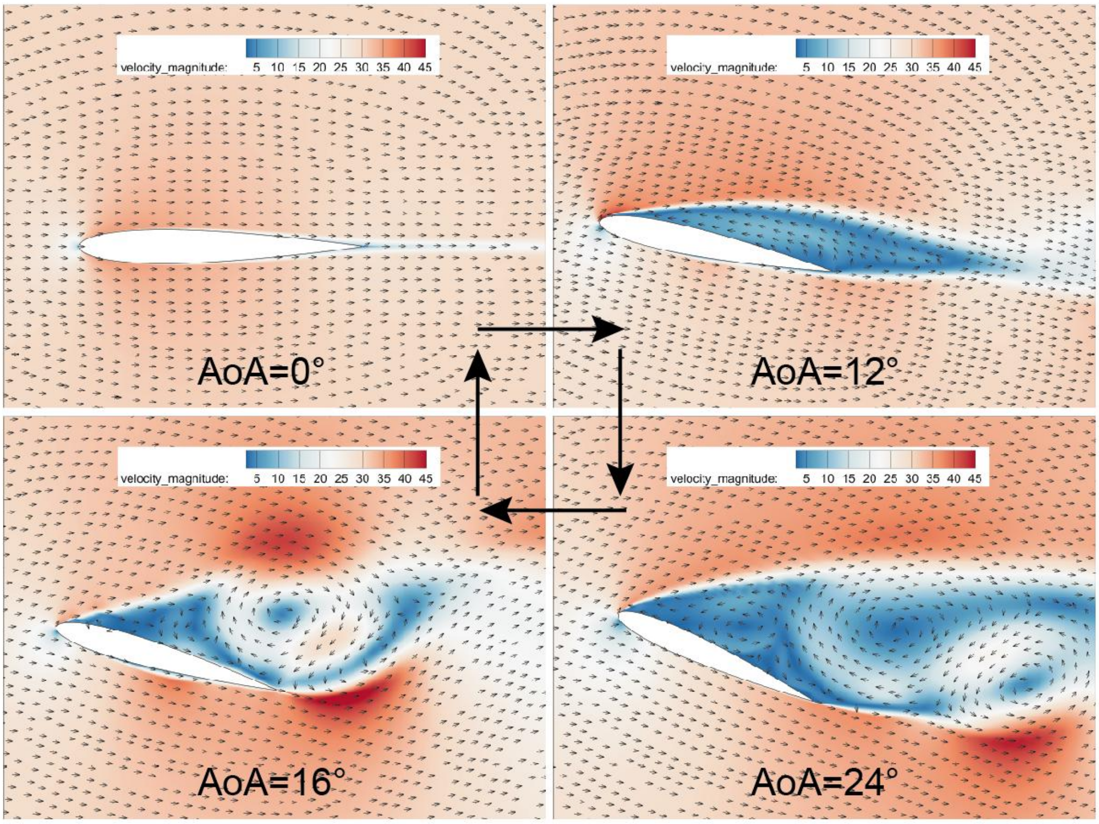

Supplementary Fig. 43 CFD simulation of the flow state around the airfoil surface when the AoA of the aircraft increased from 0° to 24°.

Ansys Fluent was used to simulate the flow direction and velocity of the incoming air around the aircraft's airfoil. NACA0012 two-dimensional airfoil was employed to simulate. The simulation was carried out at standard atmospheric pressure with an incoming flow velocity of 30 m/s, and the surface airflow flow states under the conditions of AoA=0°, 12°, 16° and 24° were calculated respectively. Set the chord length (L) of the wing to 0.2524 m, and the corresponding Reynolds number at this time was about 518369.3：

$$Re = \frac{\rho v L}{\mu} = \frac{1.225 \times 30 \times 0.2524}{1.7894 \times 10^{-5}} = 518369.3, \quad (2)$$

where ρ represented the fluid density, v represented the incoming flow velocity, and μ represented the viscosity coefficient. There was a very large velocity gradient in the

normal velocity near the wall (airfoil surface). In an ultra-small distance, since the velocity dropped from a relatively large value to the same as the wall velocity, for the calculation of the flow field in this area, a dense grid and the wall model method was used to calculate. In the simulation of this work, in order to solve the viscous sublayer region, the value of y^+ was taken as 1 (the turbulence model was SST k-omega). Since y^+ directly affected the position of the first layer grid node (the thickness of the first layer grid), the first layer grid thickness y of the model was calculated by y^+.

$$y = \frac{y^+ \mu}{U_\tau \rho}, \tag{3}$$

where, μ represented the viscosity coefficient, U_τ represented the friction velocity, and ρ represented the fluid density.

$$U_\tau = \sqrt{\frac{\tau_w}{\rho}}, \tag{4}$$

where τ_w was the wall shear stress.

$$\tau_w = \frac{1}{2} C_f U_\infty^2, \tag{5}$$

where C_f was the wall friction coefficient.

$$C_f = (2log_{10}Re - 0.65)^{-2.3}, \tag{6}$$

therefore, the mesh thickness of the first layer of this work was finally determined to be $y=1.06\times10^{-5}$ m. The flow field area was 10 times the chord length. The entire grid of the fluid adopted a structured grid, and the minimum value of the relative determinant of the grid was 0.906. The minimum grid angle was 42.84° and 143,160 grids were divided. Since the flow field on the airfoil surface was very important, in order to solve the viscous sublayer region, we used SST k-omega model as the turbulence model to calculate the turbulent kinetic energy K and the turbulent dissipation rate of this work.

$$K = \frac{3}{2}(v_{avg} I), \tag{7}$$

where, v_{avg} represented the average velocity of the flow field, and I represented the turbulence intensity.

$$I = \frac{v'}{v_{avg}} = 0.16\left(Re\right)^{-1/8}, \tag{8}$$

v' represented the velocity fluctuation, so the turbulent kinetic energy K of this simulation was 1.28.

Next, calculated the turbulent dissipation rate ε,

$$\varepsilon = C_{\mu}^{3/4}\frac{K^{3/2}}{l}, \tag{9}$$

where C_{μ} was an empirical constant, whose usually taken as 0.09, and l was the turbulent length scale (0.007 L). After calculation, the turbulent dissipation rate of this simulation was 13.59. The energy equation and k-omega model in Ansys Fluent were activated. In order to better simulate the compressible type of fluid and reflect the relationship between pressure and density, the ideal-gas equation was used for the gas simulated in this work (for high-speed compressible flow problems, usually the relationship between the physical properties of the fluid and the temperature was relatively large, this simulation simplified it, setting the thermal conductivity of the specific calorimeter to a constant value and the viscosity to sutherland). We set the boundary type of the inlet boundary to pressure far field and the boundary type of the airfoil boundary to wall. The solution method adopted implicit and Roe-FDS solution, and set turbulent kinetic energy and specific dissipation rate as second order upwind. Set number of time steps to 500, time step size to 0.002, and max iterations to 100 to calculate in this work.

Supplementary Note 9

Detailed settings and calculations of 3D CFD simulation

(1) **The creation of 3D models.** The 3D model of the steel sheet of DATSS system and aircraft wing is shown in Supplementary Fig. 44. The wing shape curve was the section curve of NACA0012. The parameters of the curve were obtained from the official website of airfoiltools (http://airfoiltools.com/). Based on the calculation amount, the wing model was finally determined to be 195 mm in chord length and 200 mm in span length. The size of the sheet body was 110 mm long, 20 mm wide and 0.4 mm thick. Its material was set as structural steel. The mounting position was 25 mm from the leading edge vertex of the wing and was centered above the wing.

The specific parameters are shown as followed: the material density was 7850 kg/m^3, Young's modulus was 2E+11 Pa, Poisson's ratio was 0.3.

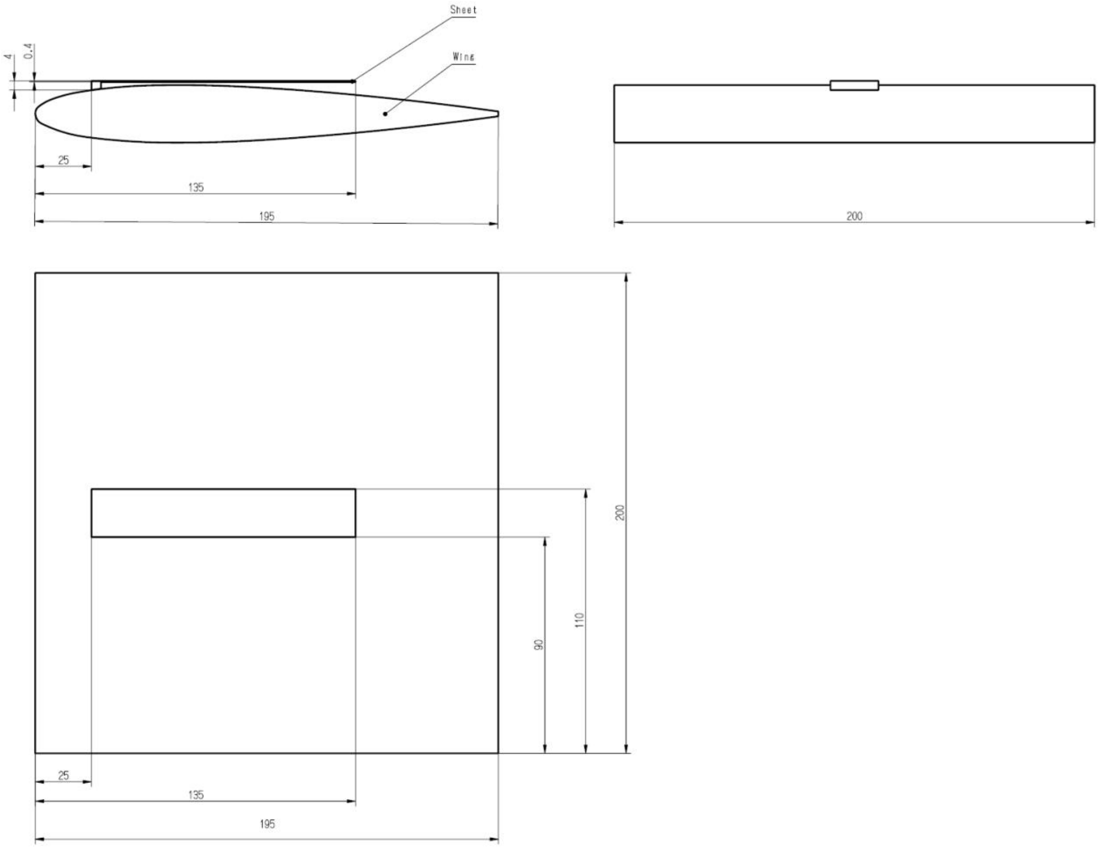

Supplementary Fig. 44 The 3D model of the steel sheet of DATSS system and aircraft wing.

(2) **CFD simulation conditions.** In order to simulate the real environment of the wing, the simulated incoming flow velocity (*v*) was set as 200 m/s and the Reynolds number (*R*e) was 2,669,889 at standard sea level temperature, y^+ was 30. The simulation calculated the motion state of the DATSS at AoA of 0° and 18° respectively. The simulation used two-way fluid-structure interaction simulation, and the transient structural module of Ansys Fluent was used for calculation (Supplementary Fig. 45).

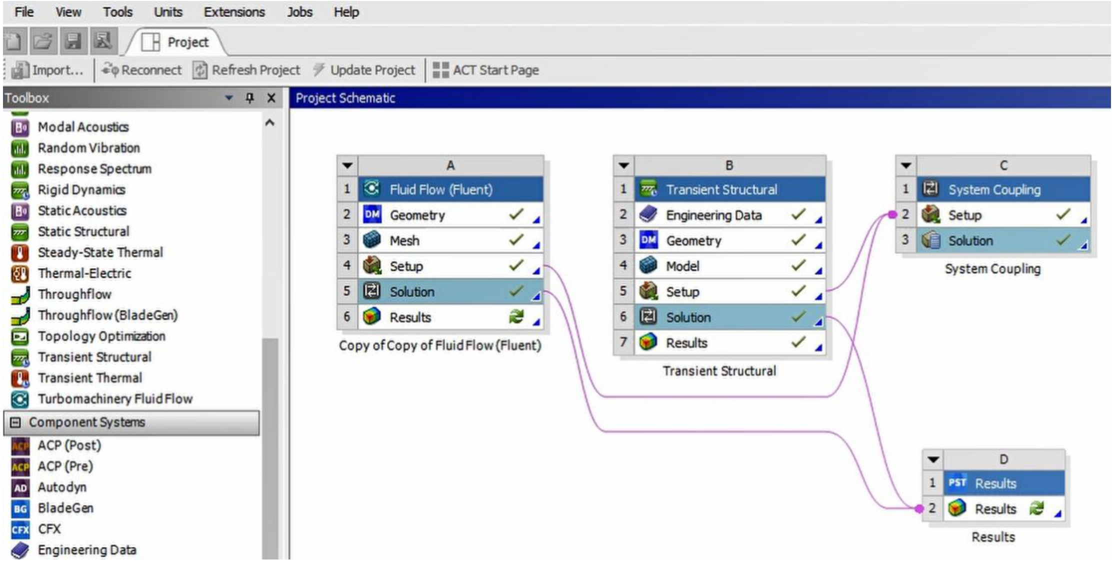

Supplementary Fig. 45 Ansys Workbench calculates flowchart.

(3) **Pre-processing of models.** The fluid domain was generated using Fluent's Geometry module, whose size was 1500 mm long, 300 mm wide and 400 mm high. The model was placed at 1/3 of the length direction, and placed symmetrically in the Y-axis and Z-axis directions. For the convenience of grid division in the later period, the fluid domain was divided into blocks based on the mesh model of the wing and steel sheet (Supplementary Fig. 46).

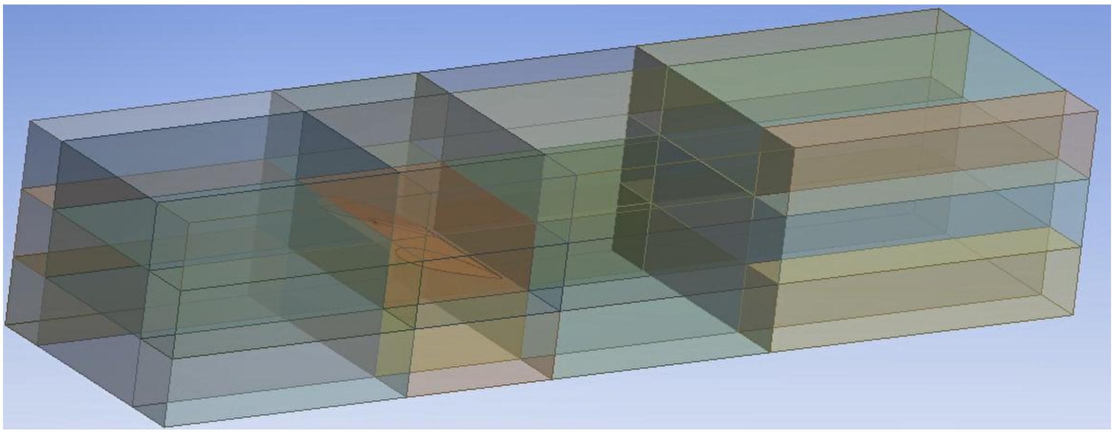

Supplementary Fig. 46 Partition of fluid domain blocks.

The Mush module of Fluent was used to generate the fluid domain grid. Entering the Mush module, the solid part was suppressed first, and the fluid domain part was generated by grid (Supplementary Fig. 47).

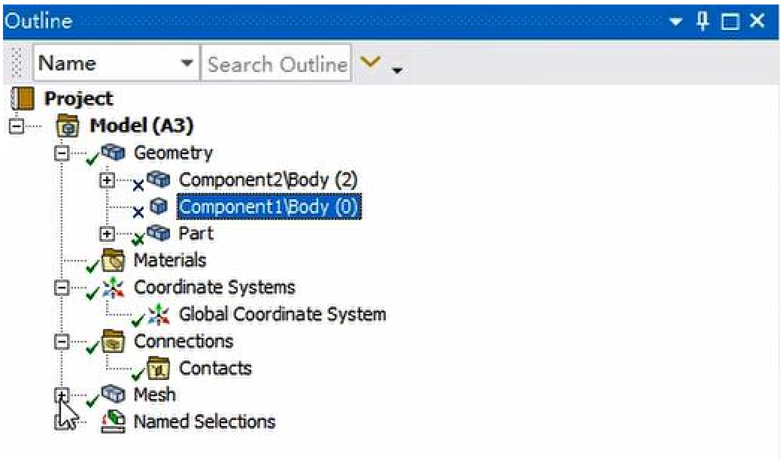

Supplementary Fig. 47 The solid part is suppressed.

In fluid grid generation, face sizing command was used to control the mesh size of steel sheet, and the element size of steel sheet was 2.0 mm. The mesh size of fluid domain was generated using body sizing command, and the element size of fluid was 10.0 mm. The resulting grids are shown in Supplementary Fig. 48 and Fig. 49.

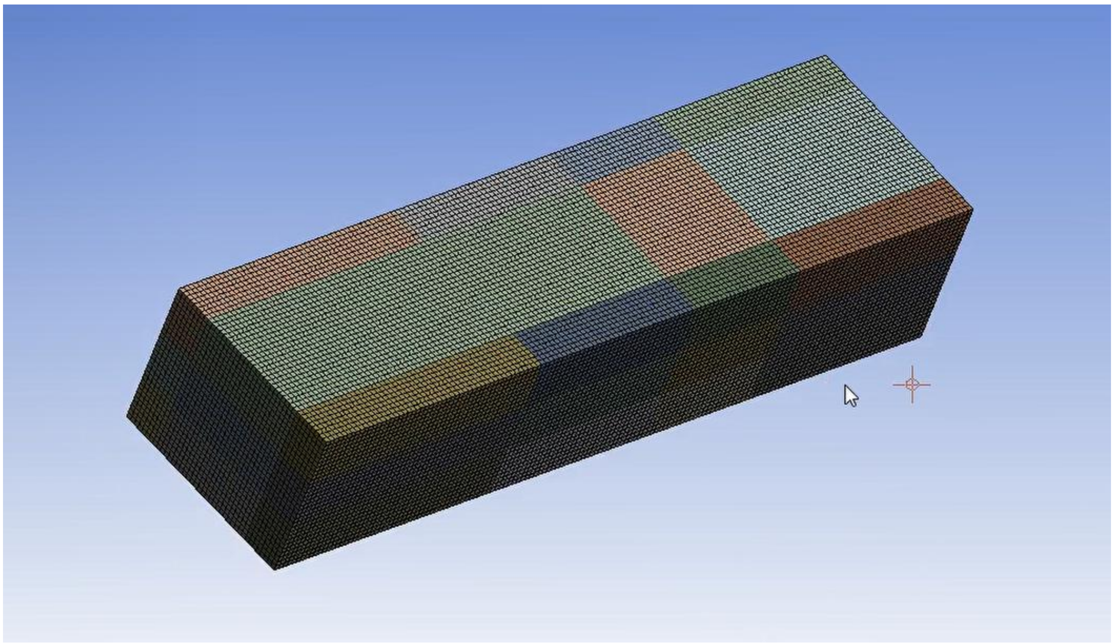

Supplementary Fig. 48 Fluid domain grid generation.

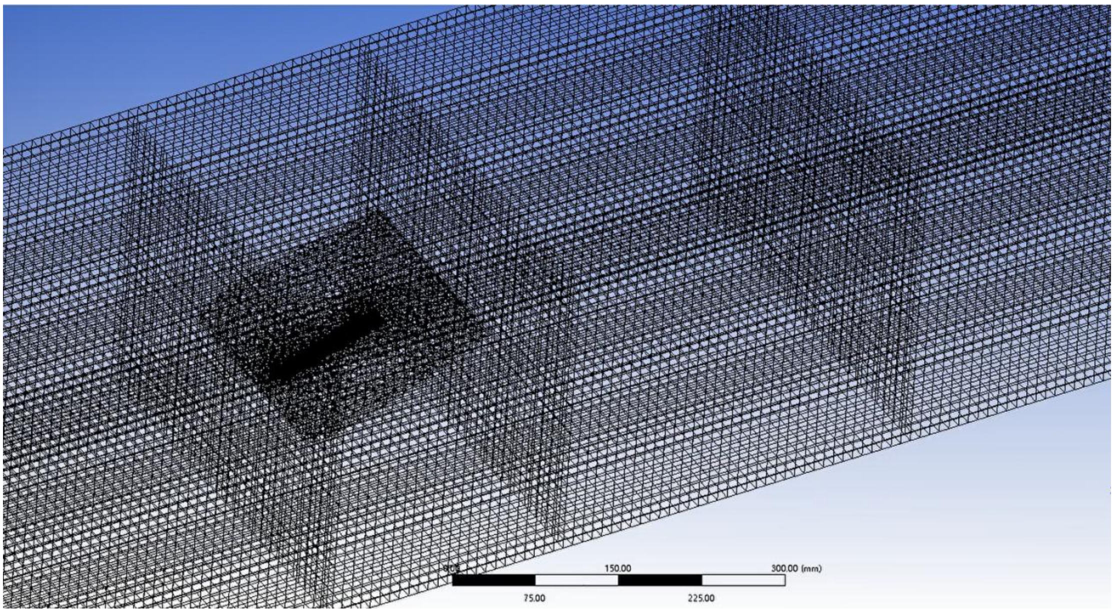

Supplementary Fig. 49 Enlarged view of 3D model mesh detail.

Create named selection was used to name the cross sections of the fluid domain so that the boundary conditions could be applied later. The corresponding surface of the leading edge of the wing was the fluid inlet surface (inset), and the corresponding surface of the trailing edge of the wing was the fluid outlet surface (outset). The upper, lower and left and right sides of the wing were set as planes of symmetry (sym1-4). Then, the wing surface (yi) and the fluid-structure interaction surface (fsiwall) of the steel sheet were set respectively.

(4) **Fluent setting.** After the grid model was imported, the general item was set. The type item of solver module was set as pressure-based, the velocity formulation item was set as absolute, and the time item was set as transient (Supplementary Fig. 50).

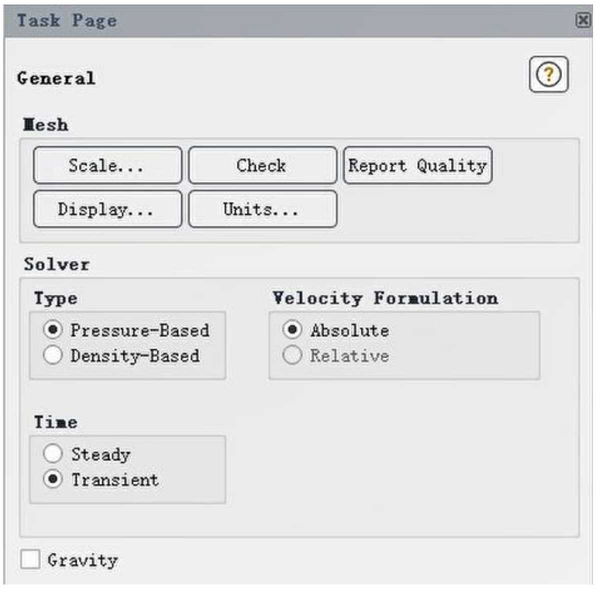

Supplementary Fig. 50 General setting.

Models setting: opened the energy equation under the models module, selected SST *k*-omega model for turbulence model, and adopted Fluent default settings for other settings (Supplementary Fig. 51).

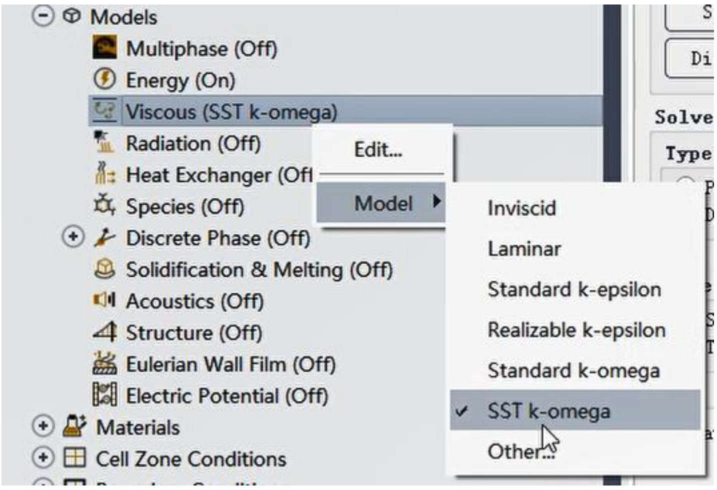

Supplementary Fig. 51 Models setting.

Materials setting: in this module, the material in the fluid domain was set to air, the density was set to ideal-gas, and the other gas parameters were set by Fluent default settings (Supplementary Fig. 52).

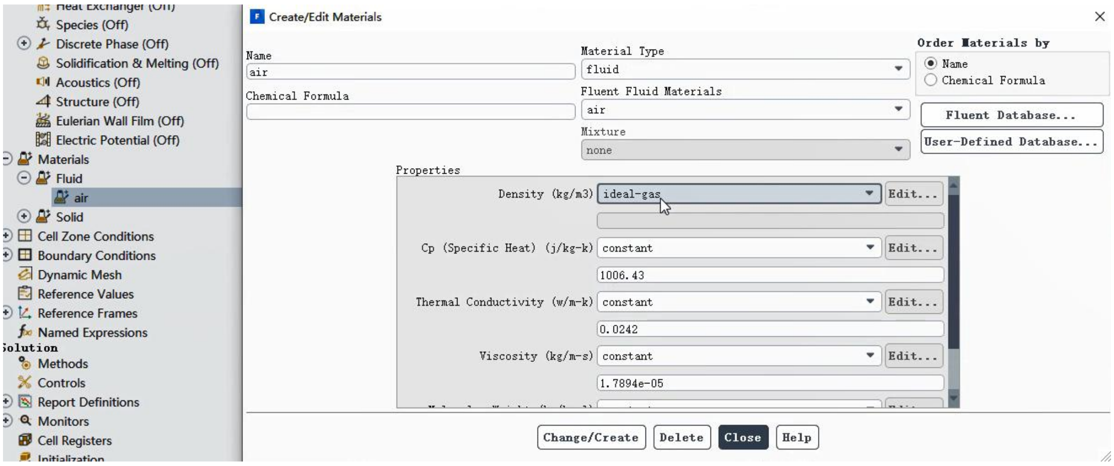

Supplementary Fig. 52 Materials setting.

Boundary conditions setting: set corresponding boundary conditions in this module, and referred to the naming rules mentioned above for specific boundary conditions. Velocity-inlet was set at the boundary of fluid inlet, where the incoming flow speed was 200 m/s, and other inlet parameters were set by default. The outlet boundary was set to the pressure outlet. The plane of symmetry (sym1-4) was set as the symmetric boundary condition, and the wing boundary (yi) and the fluid-structure interaction surface (fsiwall) were both wall boundary conditions (Supplementary Fig. 53 and 54).

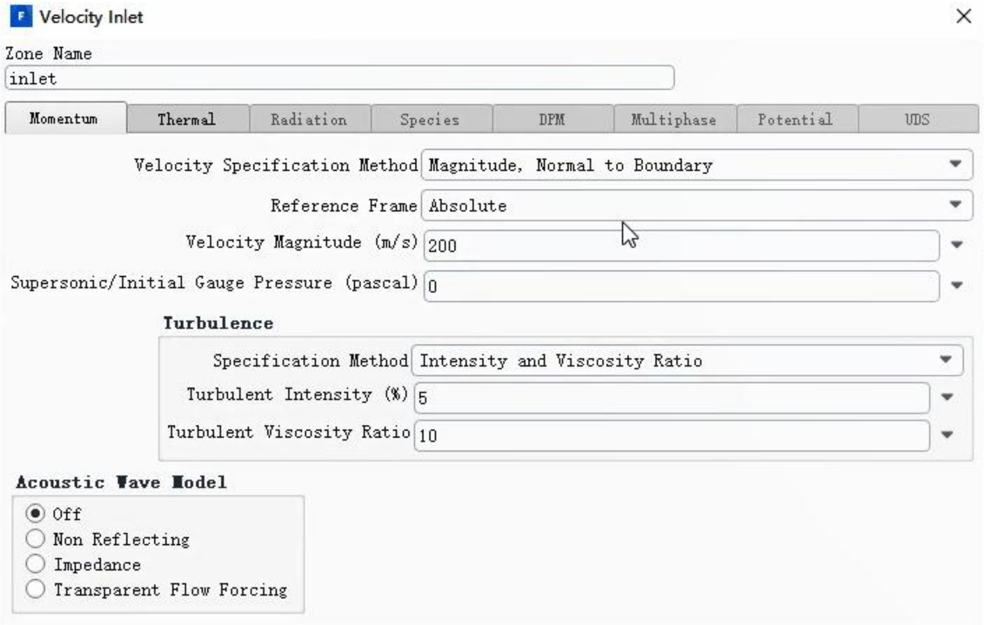

Supplementary Fig. 53 Velocity-inlet setting.

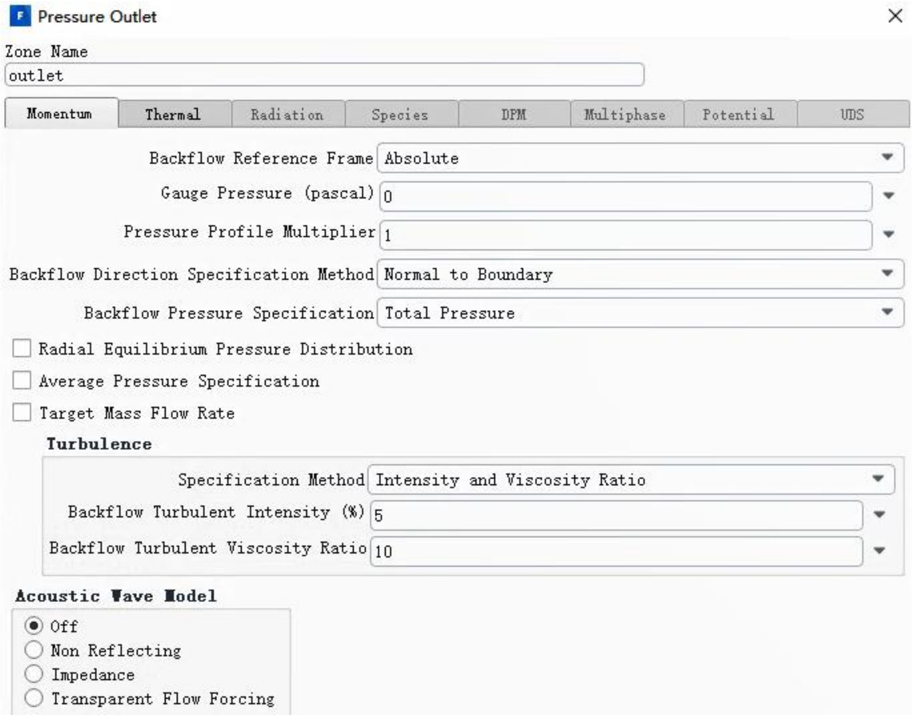

Supplementary Fig. 54 Pressure-outlet setting.

Dynamic mesh setting: in this module, set specific parameters of dynamic mesh. In mesh methods item, selected smoothing and remeshing modes, and in options item, selected implicit update, contact and detection (Supplementary Fig. 55).

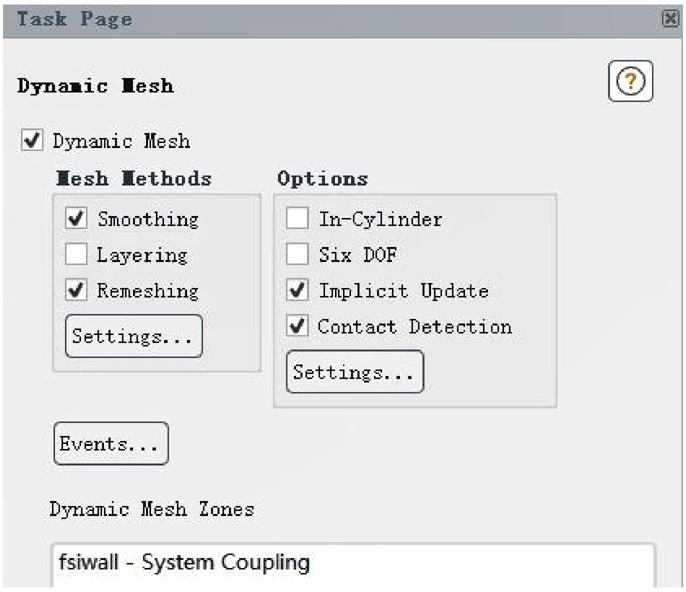

Supplementary Fig. 55 Dynamic mesh setting.

Then selected the linearly elastic solid mode from the smoothing option in the mesh method settings module, selected the local cell mode in the remeshing option, and set the size remeshing interval in the parameters to 1, that was, grid update was calculated once per iteration. Set the contact gap between yi boundary and fsiwall boundary in the contact detection of the item of options, that was, the proximity threshold was 0.1 mm. Finally, the boundary of fsiwall was created as a fluid-structure interaction system coupling (Supplementary Fig. 56-59).

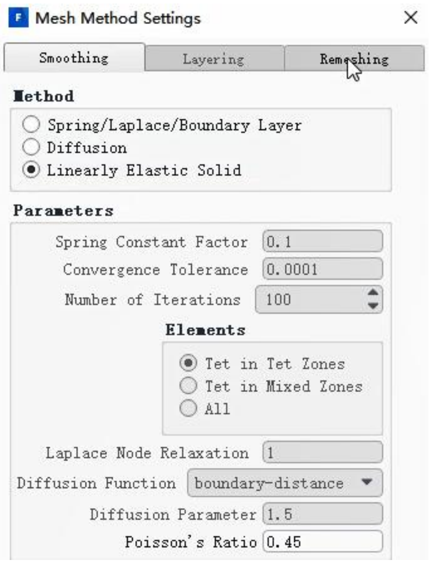

Supplementary Fig. 56 Smoothing setting.

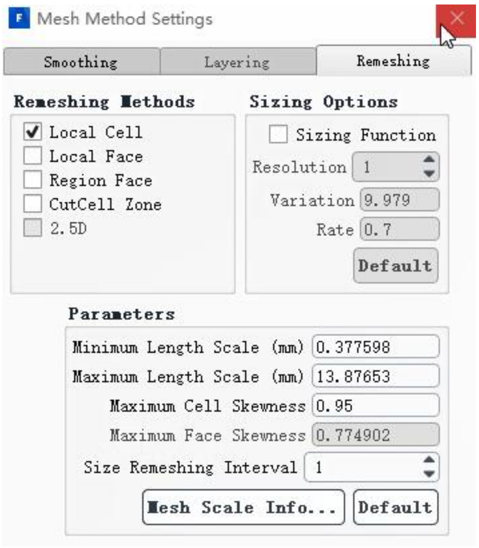

Supplementary Fig. 57 Remeshing setting.

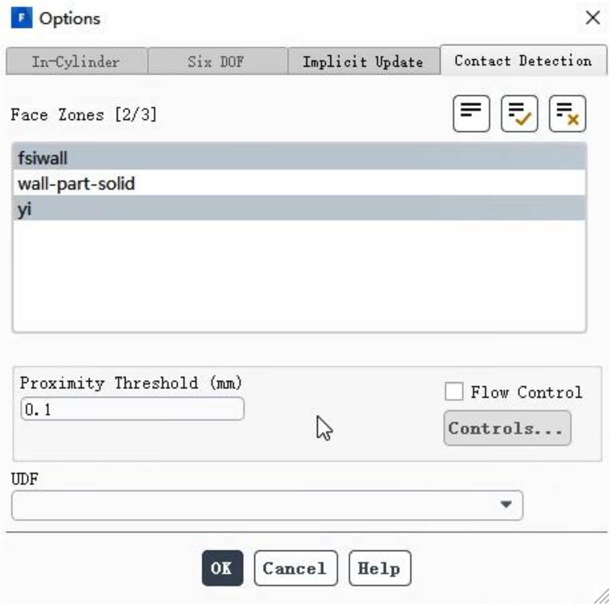

Supplementary Fig. 58 Options setting.

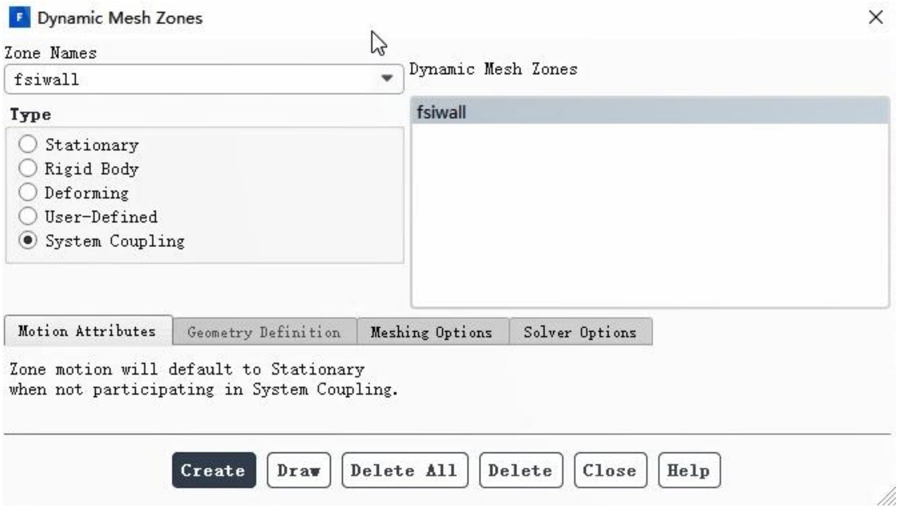

Supplementary Fig. 59 Fluid-structure interaction setting.

Solution methods setting: set the relevant parameters of the solver in this module, Supplementary Fig. 60 shows the detailed settings. **Solution initialization setting:** inlet boundary conditions were used to initialize the fluid domain calculation. The related settings and results are shown in Supplementary Fig. 61. **Solution run calculation setting:** set the time step size to 0.0001 and the number of time steps to 1. Since this simulation was a two-way fluid-structure interaction calculation, the calculation in the Fluent solver was not the actual calculation step size and calculation

step. For the setting of relevant parameters, refer to the parameter setting in the following section (Supplementary Fig. 62).

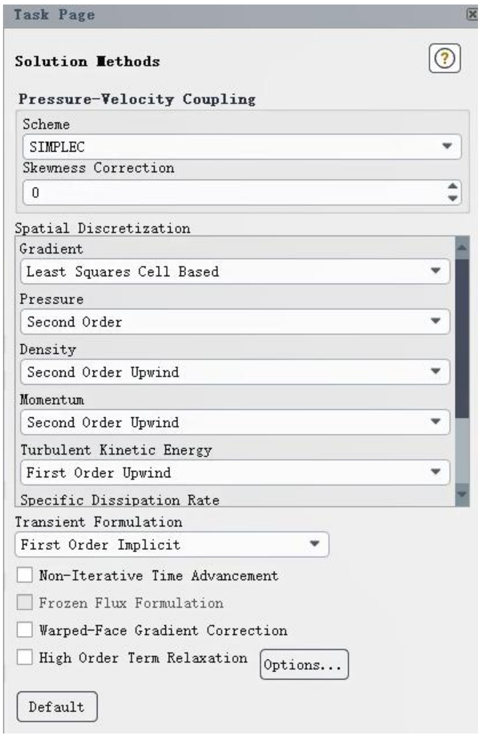

Supplementary Fig. 60 Solution methods setting.

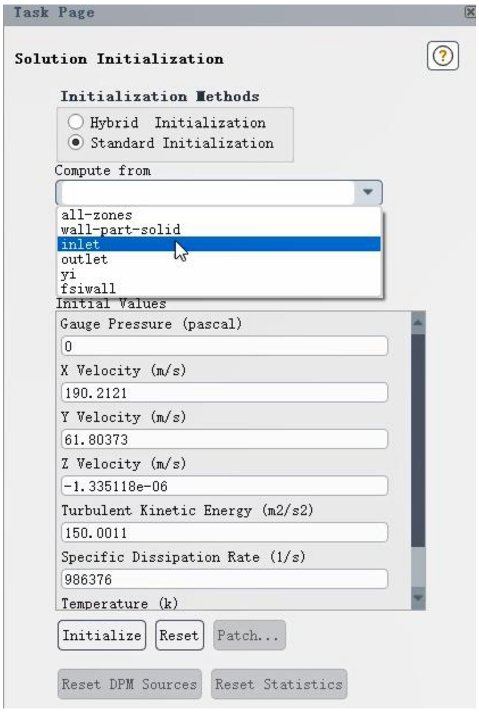

Supplementary Fig. 61 Solution initialization setting.

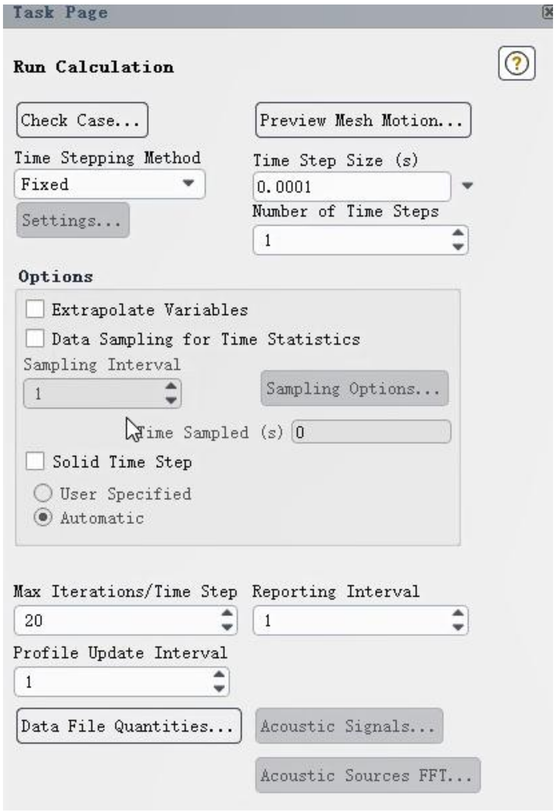

Supplementary Fig. 62 Solution run calculation setting.

(5) **Transient structural setting. Pre-processing of the model:** set the relevant parameters of the fixed part, in the pre-processing part, the model related to the fluid domain needed to be suppressed first. Materials setting: set the material parameters of the piece body, as shown in Supplementary Fig. 63.

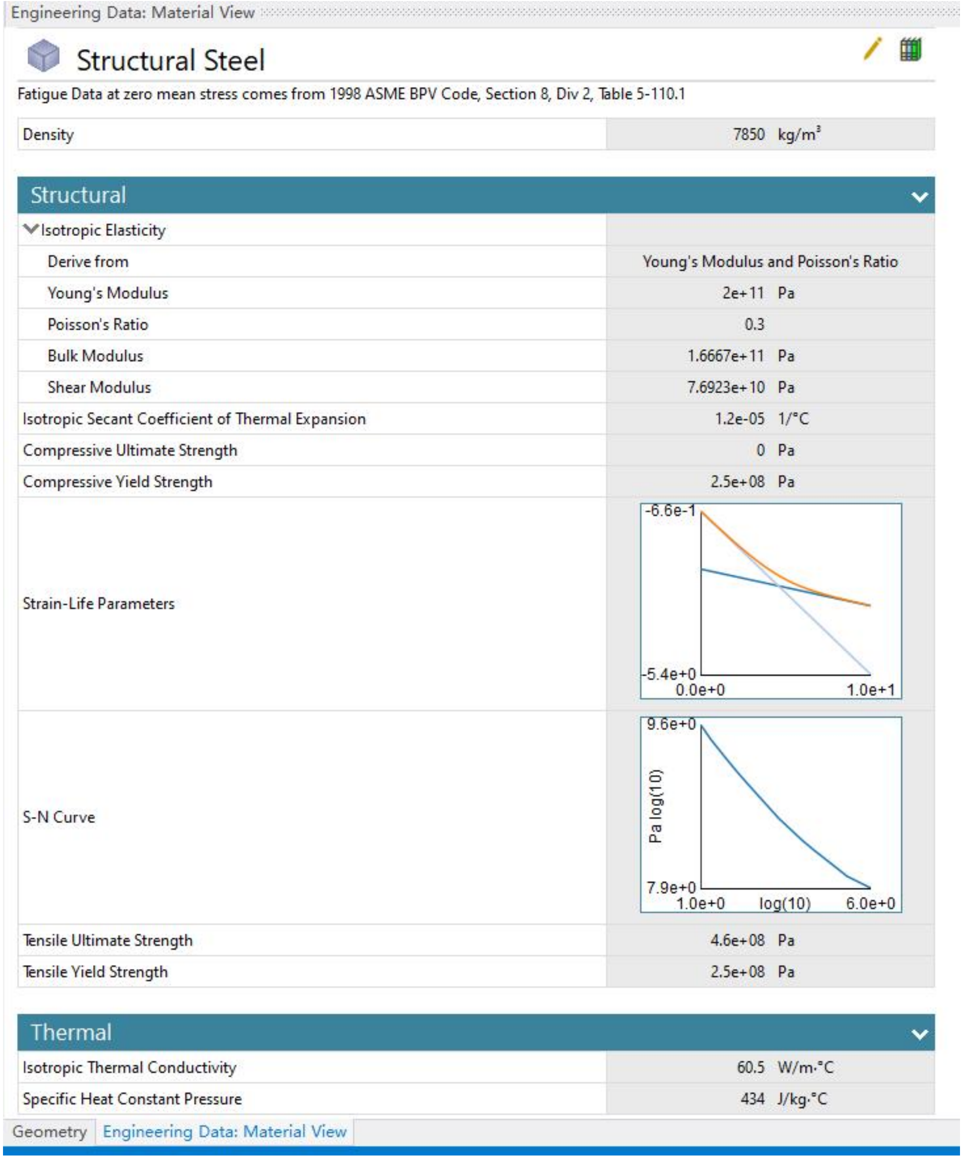

Supplementary Fig. 63 Materials setting.

Connections setting: in this part, the minimum distance between the airfoil on the wing and the steel sheet was set. The type was set to frictionless mode, and the minimum distance item "offset" was set to 0.1 mm (Supplementary Fig. 64).

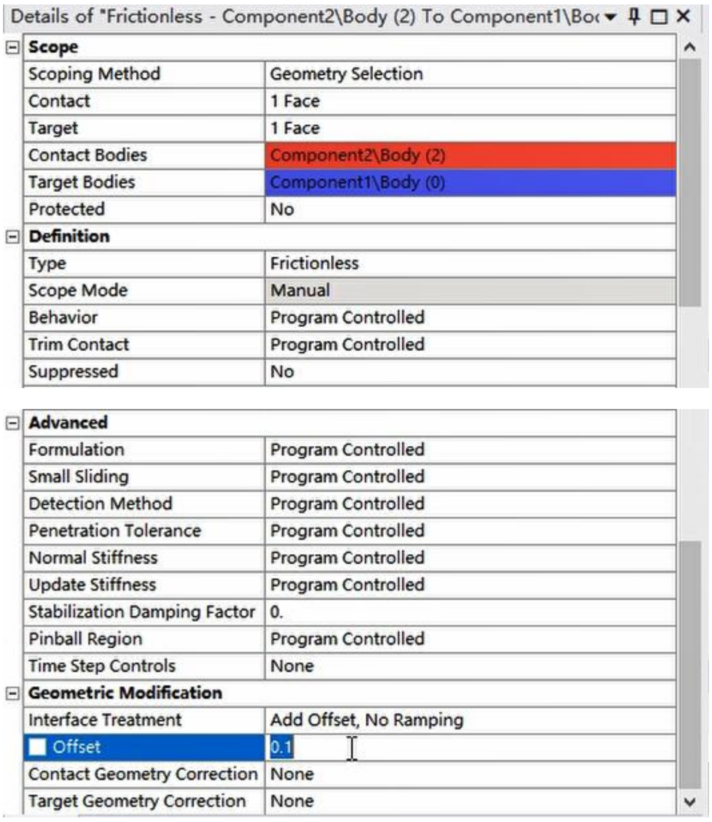

Supplementary Fig. 64 Connections setting.

Model grid generation: body sizing was used to generate the mesh of the wing and the steel sheet respectively. This simulation focused on the influence of the DATSS in the fluid domain, so a small-size mesh was generated for the steel sheet mesh, and its element size was set to 2.0 mm. For the wing mesh, the element size of the wing was set to 20.0 mm, the final grid model is shown in Supplementary Fig. 65. **Transient fixed support setting:** set the fixed constraint surface of the steel sheet, as shown in Supplementary Fig. 66. **Transient fluid solid interface setting:** six surfaces of the steel sheet were set as fluid-structure interaction surfaces, and the results were shown in Supplementary Fig. 67. **Transient fixed support setting:** set the wing surface as a fixed surface, and the result is shown in Supplementary Fig. 68.

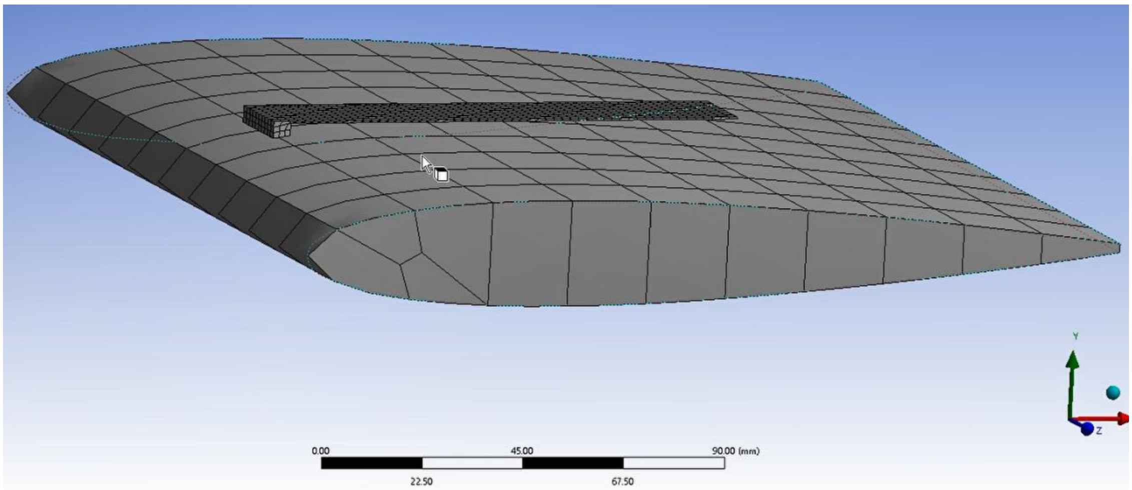

Supplementary Fig. 65 3D model grid generation diagram.

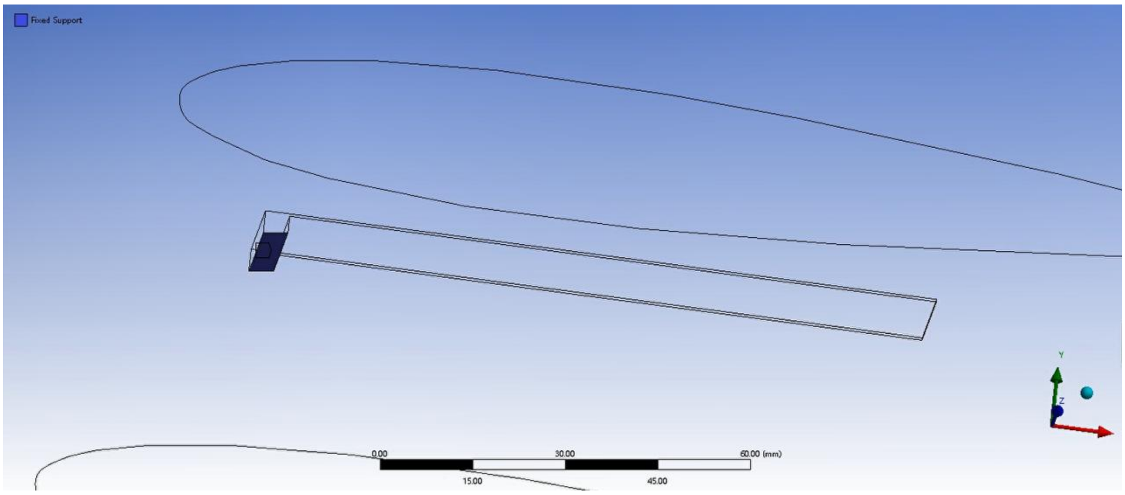

Supplementary Fig. 66 The fixed constraint surface of the steel sheet.

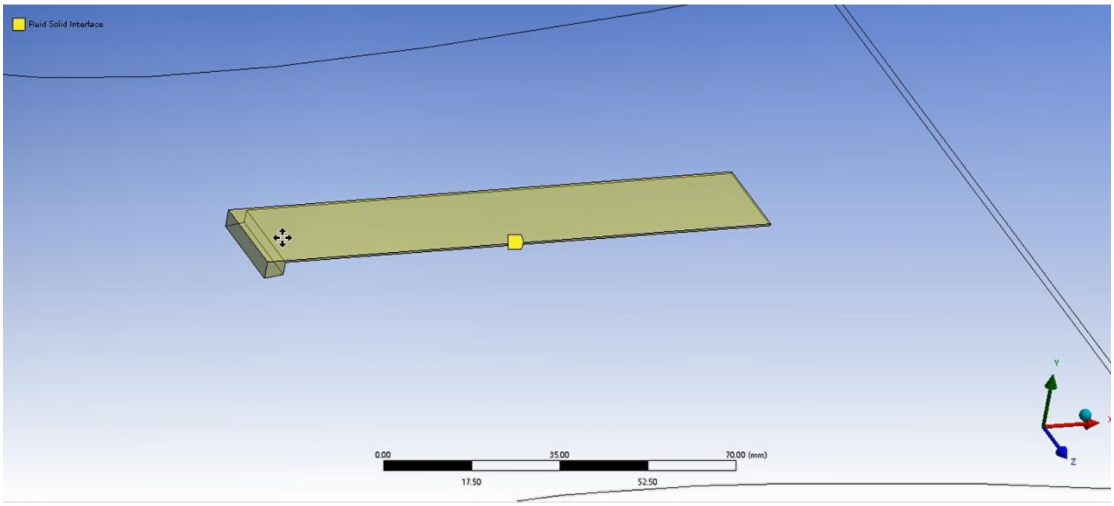

Supplementary Fig. 67 The setting of fluid-structure interaction surfaces.

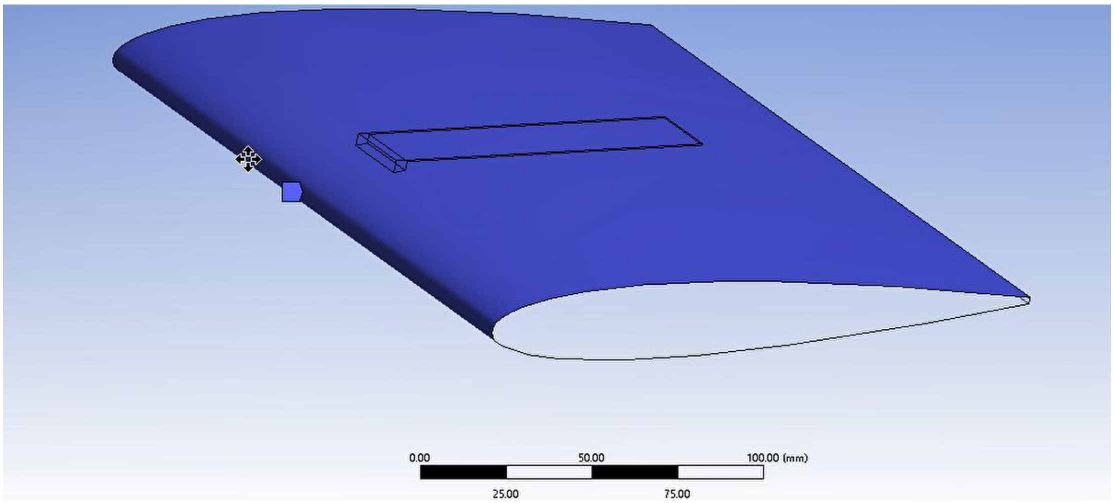

Supplementary Fig. 68 Wing surface fixed constraint setting.

(6) **System coupling setting. Analysis settings:** set the simulation calculation termination time and calculation step length. The total calculation time of AoA=18° was 1 s, and the time step was 5E-05 (the total calculation time of AoA=0° was 2 s, and the time step was the same as AoA=18°). **Data transfers setting:** set the data transfer between the steel sheet and the fluid domain, the results are shown in Supplementary Fig. 69.

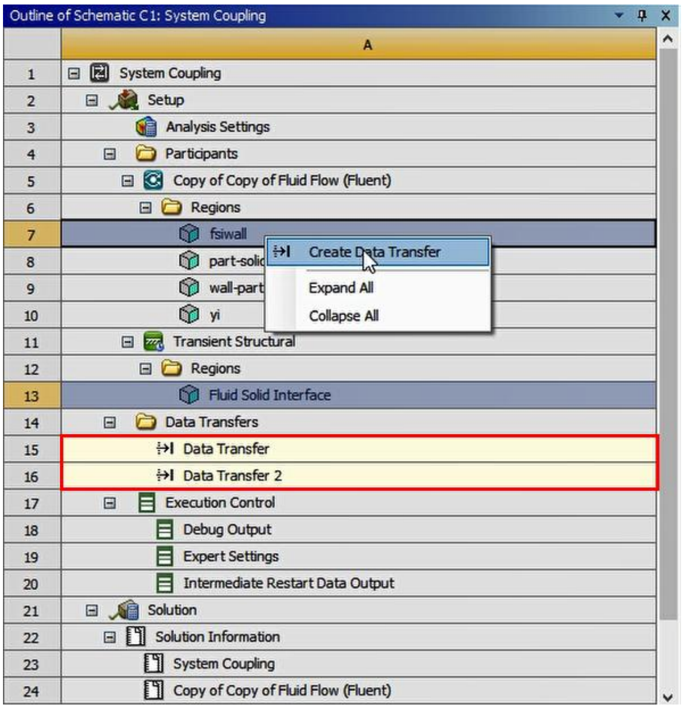

Supplementary Fig. 69 Data transfers setting.

(7) **Convergence curve**. The convergence curves calculated for AoA= 0° and 18° are shown in Supplementary Fig. 70 and 71.

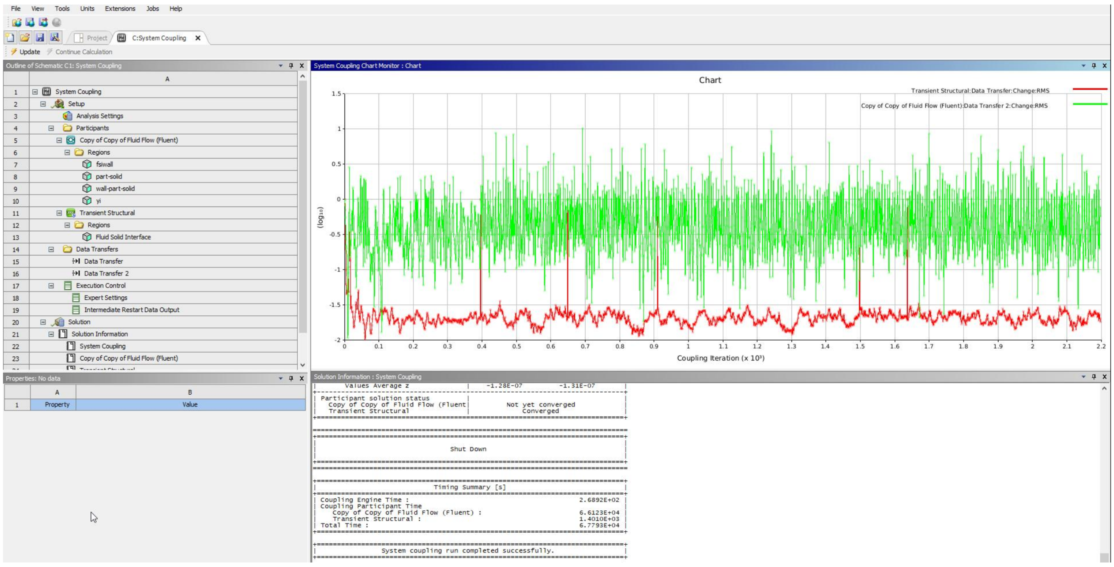

Supplementary Fig. 70 Calculated convergence curve for AoA=0°.

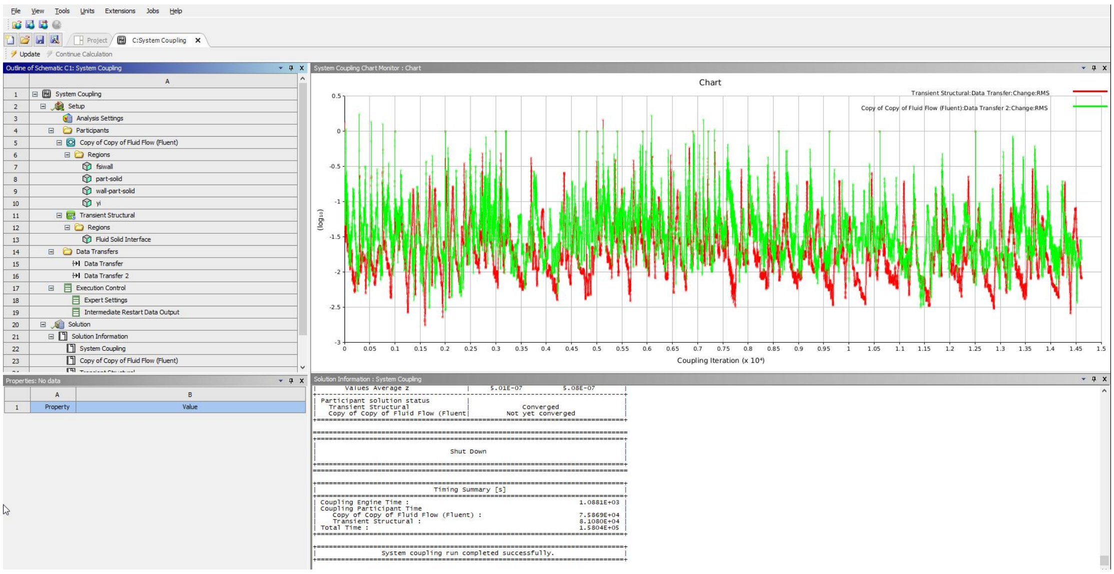

Supplementary Fig. 71 Calculated convergence curve for AoA=18°.

(8) **Post-processing of the result file.** The Results module is used for post-processing. After importing the result file, the Plane command is used to draw the display plane (Plane 1), as shown in Supplementary Fig. 72. The velocity cloud map distribution is displayed on the Plane 1 (Supplementary Fig. 73).

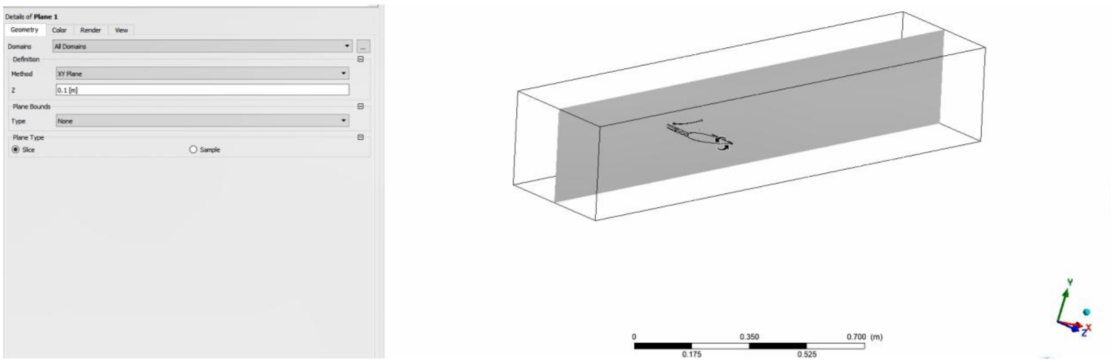

Supplementary Fig. 72 Drawing the display plane.

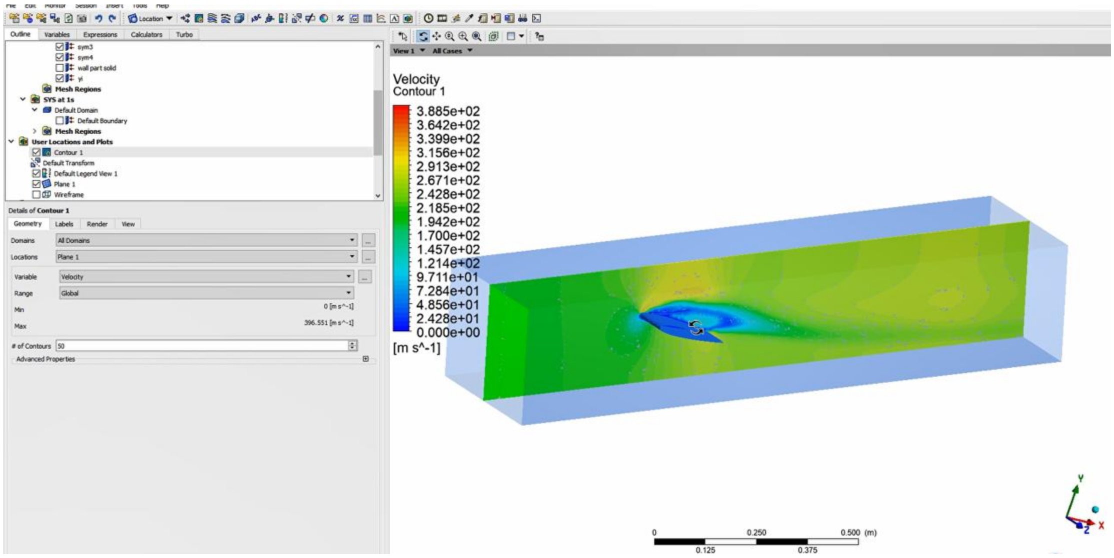

Supplementary Fig. 73 The velocity cloud map distribution.

Supplementary Note 10

DATSS system for Cessna C172S manned vehicle testing

Real aircraft flight testing requires contacting airlines, formulating flight plans, testing feasibility analysis of DATSS system, purchasing routes, installing DATSS system, installing data receivers, determining flight time and flight testing, which are complicated and difficult processes. Specific flight information is as follows (Supplementary Table 3). DATSS system test data on a real Cessna C172S is shown in Supplementary Fig. 74. The Reynolds number for cruising flight is about 5.4×10^{6}, when AoA is 16°, the Reynolds number at flight is about 4.3×10^{6}. The descending process of the T-Signal and the appearance process of the P-Signal correspond to the wind tunnel test data (Supplementary Fig. 74a). Supplementary Fig. 74b shows the

photograph of real Cessna C172S aircraft and airport information. Supplementary Fig. 74**c** exhibits the photograph of the DATSS system on the wing surface of a real Cessna C172S and inset is a takeoff test moment.

Supplementary Table 3. Flight information.

Item	Information
Flight Date	February 2, 2023 6:00 pm
Location	Heze airport, Heze city
Longitude and Latitude	Longitude:115.7° east; Latitude 35.2° north
Weather	Cloudy
Wind Direction	Northeast to South
Wind Speed	3-6 m/s
Visibility	4-10 km
Air Temperature	-3℃ - 5℃
Aircraft	Cessna C172S
Pilot	Chenglei Wang
Flight Speed	80-100 knot (41.15-51.44 m/s)
Flight Altitude	Under 900 m
AoA of Stall Flight	16° (P-Signal)
AoA of Level Flight	0° (T-Signal)
**Reynolds Number**	**~4.3×10^6 - 5.4×10^6**
Wingspan	36 feet 1 inch
Body Length	27 feet 2 inch
Tail Wingspan	11 feet 4 inch

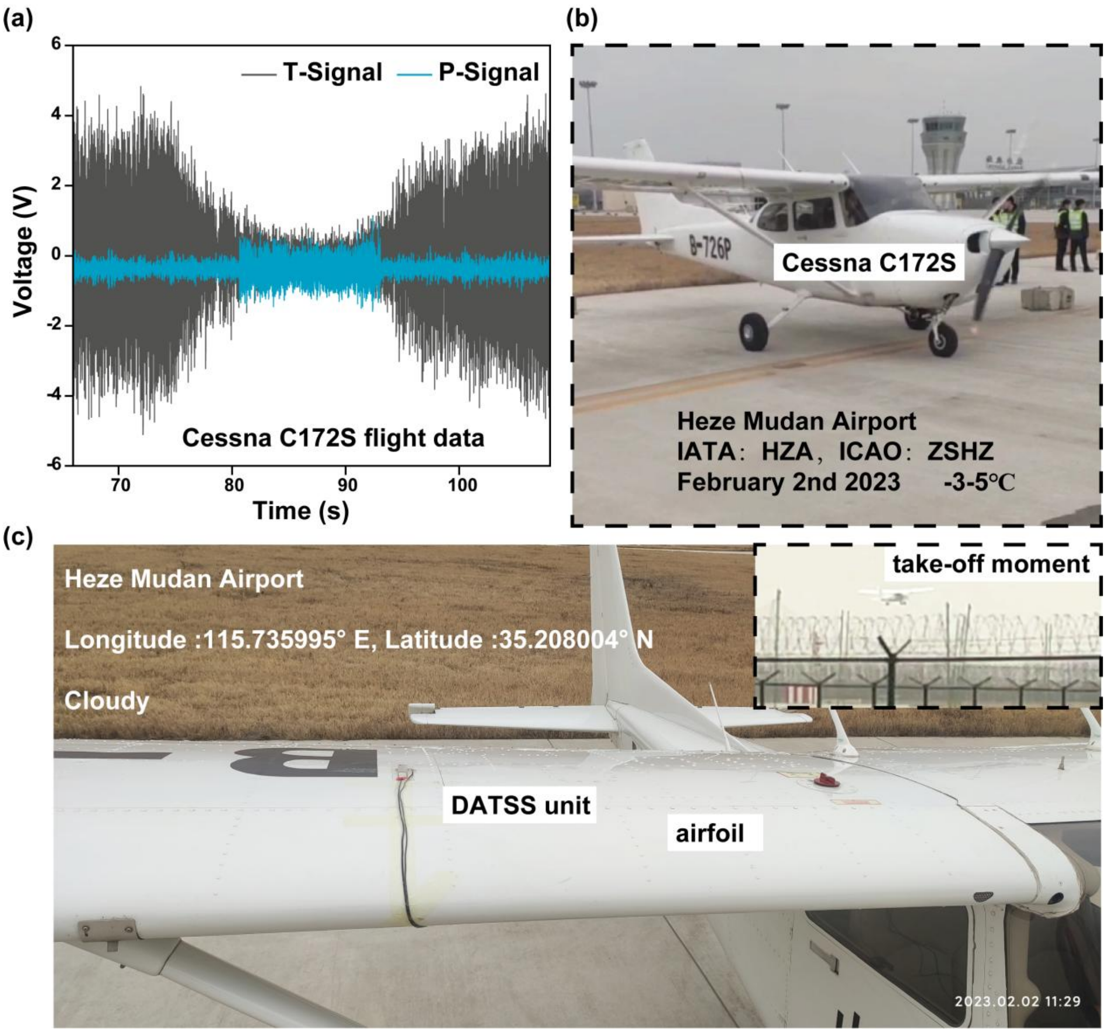

Supplementary Fig. 74 (a) The T/P-signal test of DATSS system in real Cessna C172S aircraft. (b) Photograph of real Cessna aircraft and airport information. (c) Photograph of the DATSS system on the wing surface of a real Cessna C172S and a takeoff test moment.

References

1 Bechert D*, et al.* Biological surfaces and their technological application-laboratory and flight experiments on drag reduction and separation control. *28th Fluid dynamics conference* (1997).

2 Kundu PK, Cohen IM, Dowling DR. *Fluid mechanics*. (2015).
