# Supplementary material for: Digital mapping of surface turbulence status and aerodynamic stall on wings of a flying aircraft
Source: Nat Commun. 2023 May 16;14:2792. doi: 10.1038/s41467-023-38486-6 (PMC10188437; doi:10.1038/s41467-023-38486-6)
Supplement: Supplementary file 3 — Description of Additional Supplementary Files [file 41467_2023_38486_MOESM3_ESM.docx]

File Name: Supplementary Video 1

Description: CFD at AoA=24°

File Name: Supplementary Video 2

Description: CFD at AoA=20°

File Name: Supplementary Video 3

Description: CFD at AoA=16°

File Name: Supplementary Video 4

Description: CFD at AoA=12°

File Name: Supplementary Video 5

Description: CFD at AoA=0°

File Name: Supplementary Video 6

Description: DATSS in wind tunnel

File Name: Supplementary Video 7

Description: 3D CFD of DATSS
